# Reactions of R_3_PNNPR_3_ Species with Boranes: Classical Adducts, FLPs, and Radical Cations

**DOI:** 10.1002/anie.202503331

**Published:** 2025-03-18

**Authors:** Vaibhav Bedi, Andrew Niles L. Ocampochua, Zheng‐Wang Qu, Stefan Grimme, Douglas W. Stephan

**Affiliations:** ^1^ Department of Chemistry University of Toronto 80 St. George St Toronto ON M5S3H6 Canada; ^2^ Mulliken Center for Theoretical Chemistry Clausius Institut für Physikalische und Theoretische Chemie Rheinische Friedrich‐Wilhelms‐Universität Bonn Beringstrasse 4 53115 Bonn Germany

**Keywords:** Frustrated lewis pair, Frustrated radical pair, Lewis acid‐adduct, Radical cation

## Abstract

Although the nature of *bis*‐phosphazine species is of much interest, there are few reports of their reactivity. Herein, we show that the *bis*‐phosphazine species Ph_3_PNNPPh_3_ **1** react with Lewis acids to give the Lewis acid adducts Ph_3_PNN(B(C_6_F_5_)_3_)PPh_3_ **3** and Ph_3_PN(BF_3_)N(BF_3_)PPh_3_ **4**. Compound **1** also generates a frustrated Lewis pair (FLP) in the presence of BPh_3_ and thermolysis of **1**/BPh_3_, or **3** at 80 °C released N_2_ while **4** was stable at 80 °C. In contrast, the species Cy_3_PNNPCy_3_ **2** reacted with B(C_6_F_5_)_3_ to effect single electron transfer affording a frustrated radical pair (FRP). Independent reactions of **1** and **2** with [Cp_2_Fe][BF_4_] gave the corresponding radical salts [R_3_PNNPR_3_]^•+^[BF_4_] (R = Cy **5b** Ph **6**). Structural and computational data show the N─N bond is strengthened in the *bis*‐phosphazine radical cation.

## Graphical abstract

Depending on the substituents, *bis*‐phosphazine species R_3_PNNPR_3_ (R = Ph **1**, Cy **2**) react with boranes to give Lewis acid–base adducts, generate frustrated Lewis pairs (FLP), or effect single electron transfer affording a frustrated radical pair (FRP). The B(C_6_F_5_)_3_ and BF_3_ adducts of **1** and the radical salts [R_3_PNNPR_3_]^•+^[BF_4_] (R = Cy **5b** Cy **6**) were obtained and studied structurally and computationally.

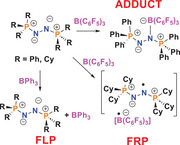

Carbon‐based N_2_ species, including diazomethanes, diazirenes, and diazonium cations, are widely used reagents, while exciting new reactivity is emerging from diazo‐ylide derivatives recently reported by the Hansmann^[^ ^1^ ^]^ and Gessner^[^ ^2^ ^]^ groups. In contrast, the reactivity of N_2_‐derivatives of other s‐ and p‐block species is much less studied. In 1978, Klemperer and coworkers described the Lewis acid‐N_2_ adduct (N_2_)BF_3_ (Figure 1a) that was generated via supersonic expansion at 600 torr and 170 K.^[^ ^3^ ^]^ In 1992, we characterized a clathrate‐like salt containing the highly unstable cation [((THF)_2_Li)_2_(μ‐N_2_)]^2+^, in which N_2_ is bound between two lithium cations (Figure 1b).^[^ ^4^ ^]^ Although Appel reported the *transoid bis*‐phosphazine species (Ph_3_P)_2_N_2_ (Figure 1c) in 1964,^[^ ^5^ ^]^ it was in 2013 that Frenking and coworkers^[^ ^6^ ^]^ described this species as two phosphine donors interacting with a doubly Lewis acidic N_2_‐unit. This description was supported by both computations and the thermal release of N_2_ affording PPh_3_ at 215 °C; however, this view was controversial.^[^ ^7^, ^8^ ^]^ In disputing this description, Himmel showed that the related carbene derivatives (C(NMe_2_)_2_)_2_(N_2_) and (C_3_H_4_(NMe)_2_)_2_(N_2_) were thermally robust, being purified by sublimation (Figure 1d).^[^ ^9^, ^10^ ^]^

**Figure 1:**
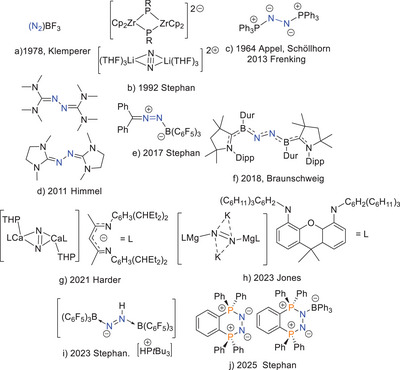
Examples of interactions of main group species with N_2_ fragments.

In 2017, we reported the species Ph_2_CN_2_B(C_6_F_5_)_3_^[^ ^11^ ^]^ prepared via reaction of borane with the diazomethane (Figure 1e). Its formation suggested that N_2_ capture could result from the simultaneous interactions with both a donor and an acceptor. Indeed, the next year, Braunschweig and coworkers^[^ ^12^ ^]^ demonstrated the capture of N_2_ by a monovalent borylene species, which is both as an electron acceptor and donor (Figure 1f). This seminal finding has led to the unveiling of related systems that couple N_2_ molecules under reductive conditions,^[^ ^13^, ^14^ ^]^ and to reduce N_2_ to ammonium chloride.^[^ ^15^ ^]^

Harder and coworkers described the synthesis of a Ca(II) N_2_^2−^ species of the form LCa(N_2_)CaL supported by a bulky β‐diketiminate ligand in 2021 (Figure 1g).^[^ ^16^ ^]^ This species was protonated to give diazene (N_2_H_2_), which disproportionated to hydrazine and N_2_. In 2023, Jones and coworkers described the reduction of a bulky magnesium(II) diamide affording a masked Mg(I) radical incorporating reduced N_2_ (Figure 1h).^[^ ^17^ ^]^ Also recently, we described the *bis‐*borane adducts of diazene (N_2_H_2_) and hydrazine (N_2_H_4_)^[^ ^18^ ^]^ (Figure 1i) and effected deprotonation to give the anionic adducts [((C_6_F_5_)_3_B)_2_(N_2_H_3_)]^−^ and [((C_6_F_5_)_3_B)_2_(N_2_H)]^−^. Attempts to remove the final hydride with Me_3_SiOTf from the latter species readily evolved N_2_ affording free borane and Me_3_SiH. Collectively, these results illustrate the instability of Lewis acid‐N_2_ interactions.

Turning our focus to systems involving the interaction of N_2_ with Lewis donors, we recently prepared the *cisoid bis*‐phosphazine species [C_6_H_4_(PPh_2_)_2_(μ‐N_2_)] (Figure 1j).^[^ ^19^ ^]^ This species undergo photolysis to give nitrene insertion into a P─C bond affording [C_6_H_4_(P(NPh)Ph)(PPh_2_)(μ‐N)], whereas thermolysis in the presence of a Lewis acid afforded the clean release of N_2_.

Continuing our studies of *bis*‐phosphazines, herein we prepared the known *bis*‐phosphazine species Ph_3_PNNPPh_3_ **1** employing minor modifications of the published method (Scheme 1)^[^ ^5^ ^]^ to obtain **1** in 30% isolated yield. Adapting this methodology, we oxidized Cy_3_P with C_2_Cl_6_ to isolated Cy_3_PCl_2_ in 95% yield as evidenced by the clean ^31^P resonance at δ = 107.6 ppm. Subsequent reaction with N_2_H_4_ and deprotonation with *t*BuOK afforded the species Cy_3_PNNPCy_3_ **2** in 40% yield (Scheme 1). This compound showed a ^31^P resonance at δ = 27.2 ppm. Single crystals of **2** were obtained from a mixture of *o*‐C_6_H_4_F_2_: hexane (1:5) at −30 °C. A single crystal X‐ray diffraction study of **2** confirmed the formulation (Figure 2).^[^ ^20^ ^]^ The two independent molecules in the asymmetric unit revealed P─N distances of 1.5886(18) Å, 1.5833(18) Å, 1.5920(18) Å, and 1.5815(18) Å with N─N distances of 1.5122(19) Å, and 1.5038(19) Å, respectively. The two phosphorus atoms adopt a pseudo‐*transoid* disposition with N─N─P angles of 108.17(11)°, 109.47(12)°, 109.12(11)°, and 108.81(10)°. This orientation of the phosphine fragments and the P─N distances in **2** is similar to that reported for **1** (N‐N‐P: 107.1(1)°, P‐N: 1.582(1) Å, N‐N: 1.497(2) Å)^[^ ^6^ ^]^ although the N─N distance is slightly longer in **2**.

**Scheme 1:**
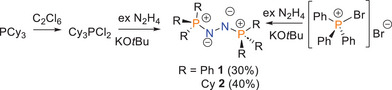
Synthesis of R_3_PNNPR_3_, (R = Ph **1**, Cy **2**).

**Figure 2:**
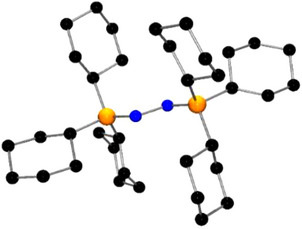
POV‐ray depictions of one of the two molecules in the asymmetric unit of **2**. Hydrogen atoms are omitted for clarity. C: black, N: blue, P: orange.

The reaction of **1** with B(C_6_F_5_)_3_ was performed at −30 °C and monitored by nuclear magnetic resonance (NMR) spectroscopy. This revealed the formation of a new species **3** as evidenced by the ^11^B NMR signal at δ = ‐5.3 ppm and ^31^P signals at δ = 17.5 ppm and δ = 31.2 ppm with a P─P coupling constant of 22 Hz. This, in addition to the ^19^F signals at δ = ‐134.9, ‐161.4, and ‐165.5 ppm, was consistent with the formation of the Lewis acid–base adduct, Ph_3_PNN(B(C_6_F_5_)_3_)PPh_3_ **3** (Scheme 2). This formulation was further confirmed via an X‐ray crystallographic study (Figure 3).^[^ ^18^ ^]^ The binding of one of the N atoms to the boron gave rise to a B─N bond length of 1.604(2) Å. The proximal P─N bond length was 1.6391(13) Å. Whereas the distal P─N was 1.5845(11) Å, the N─N distance in **3** was found to be 1.4742(16) Å. This N─N distance is significantly shorter than that reported in the parent species **1** (1.497(2) Å).^[^ ^6^ ^]^

**Scheme 2:**
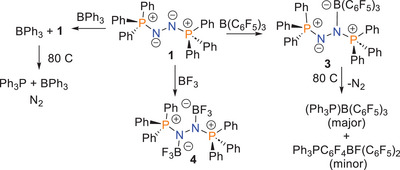
Reactivity of **1** with boranes.

**Figure 3:**
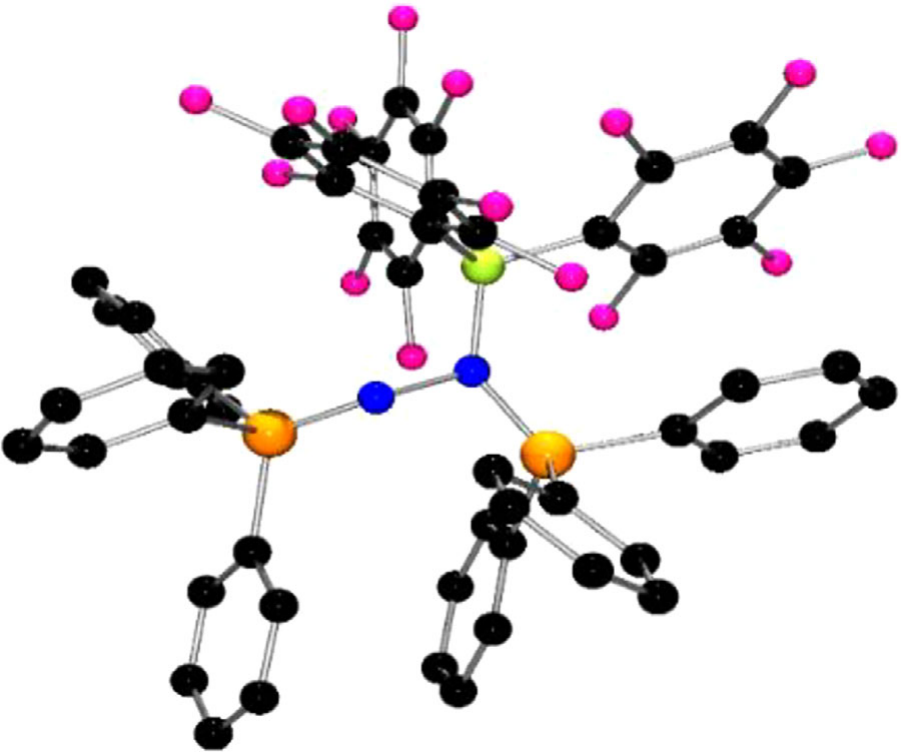
Persistence of vision (POV‐ray) depictions of **3**. Hydrogen atoms are omitted for clarity. C: black, N: blue, P: orange, B: yellow‐green, F: pink.

The analogous reaction of **1** with excess BF_3_ was performed at room temperature yielded the new species **4**. This was formulated as Ph_3_PN(BF_3_)N(BF_3_)PPh_3_ (Scheme 2) based on the ^11^B NMR signal at δ = ‐1.2 ppm, the ^19^F signal at δ = ‐151.8 ppm and the ^31^P resonance at δ = 46.3 ppm. This proposition was confirmed via an X‐ray crystallographic study (Figure 4).^[^ ^18^ ^]^ The molecule **4** is C_2_ symmetric with half of the molecule in the unit cell. The comparatively small Lewis acid BF_3_ binds to the N atoms with B─N bond lengths of 1.579(5) Å. The corresponding P─N bond length is 1.654(3) Å, whereas the N─N distance in **4** was determined to be 1.456(5) Å.

**Figure 4:**
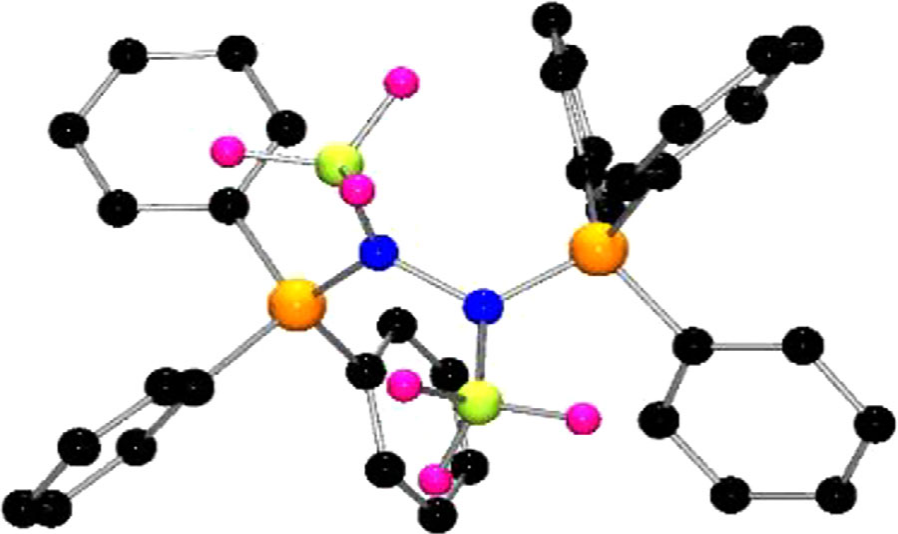
POV‐ray depictions of **4**. Hydrogen atoms are omitted for clarity. C: black, N: blue, P: orange, B: yellow‐green, F: pink.

Efforts to form a corresponding adduct with BPh_3_ revealed no reaction inferring that the mixture behaves as a frustrated Lewis pair (FLP). Heating the mixture of **1** and BPh_3_ to 80 °C, resulted in a color change from dark red to colorless. Spectral data showed production of PPh_3_ in the presence of BPh_3_, as evidenced by the ^31^P and ^11^B NMR signals at ‐6 and 60 ppm, respectively. These data are consistent with the loss of N_2_ from **1**. In a similar fashion, **3** was heated to 80 °C for 16 h. Concentration of the resulting reaction mixture afforded crystals of the known adduct Ph_3_PB(C_6_F_5_)_3_^[^ ^21^ ^]^ whereas NMR spectroscopy on the mother liquor showed the presence of unreacted **3**, PPh_3_, and the *para‐*attack product Ph_3_PC_6_F_4_BF(C_6_F_5_)_2_.^[^ ^22^ ^]^ These thermal reactions at the reduced temperature (80 °C) compared to the reported thermolysis of **1** (215 °C)^[^ ^6^ ^]^ infer that the interaction of the Lewis acid to **1** facilitates the liberation of N_2_. This view is consistent with similar observations recently reported for the reactions of C_6_H_4_(Ph_2_PN)_2_ with boranes.^[^ ^19^ ^]^

The corresponding reaction of **2** with B(C_6_F_5_)_3_ results in an immediate color change to dark olive‐green. Although this solution was NMR silent, it gave rise to a multiplet in the electron paramagnetic resonance (EPR) spectrum (see Supporting Information).^[^ ^23^ ^]^ Spectral simulation gave good agreement with a *g* value of 2.002, and hyperfine couplings of *A*_N_ = 20 G and *A*_P_ = 56 G. These data suggest single electron transfer from **2** to the borane, generating the radical cation [Cy_3_PNNPCy_3_]^•+^ **5a** (Scheme 3). The corresponding radical anion [B(C_6_F_5_)_3_]^•−^ was not observed; however, it is known to be short lived at room temperature.^[^ ^24^ ^]^ Nonetheless, this observation suggests the steric demands in **2** disfavor adduct formation prompting single electron transfer yielding a sterically frustrated radical pair (FRP).

**Scheme 3:**
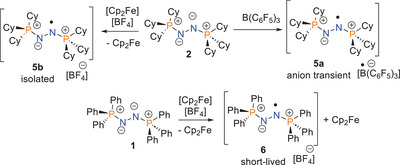
Generation of radical cations of **1** and **2**.

To further probe the stability of the radical cation, the reaction of **2** with [Cp_2_Fe][BF_4_] was performed. This generated a dark olive‐green solution similar to that seen for **5a** (Scheme 3). EPR data showed a clean and well‐resolved eleven‐line resonance, whereas the simulation gave an excellent agreement with a *g* value to 1.999 and N‐ and P‐hyperfine couplings (*A*_N_ and *A*_P_) of 20 G and 56 G, respectively (Figure 5a). In this case the product was formulated as [Cy_3_PNNPCy_3_]^•+^[BF_4_]^−^ **5b**. Blue–green crystals of **5b** were isolated from *o*‐C_6_H_4_F_2_ /hexane and a subsequent crystallographic study^[^ ^18^ ^]^ confirmed the formulation (Figure 6). The radical cation showed elongated P─N bond distances of 1.6689(13) Å and 1.6652(13) Å whereas the N─N bond distance was dramatically shortened to 1.3626(18) Å, consistent with the presence of the cationic charge.

**Figure 5:**
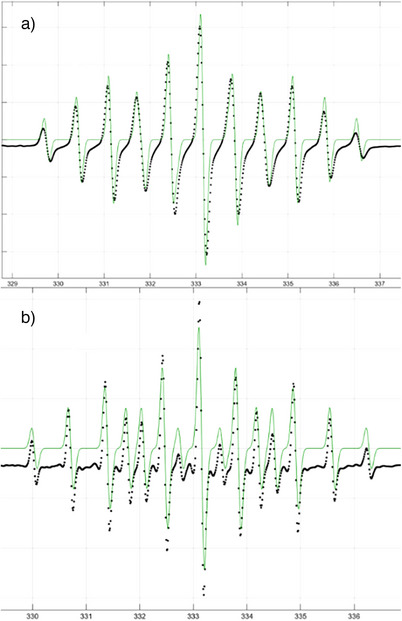
EPR spectra of a) **5b** b) **6**. Experimental (black), simulation (green).

**Figure 6:**
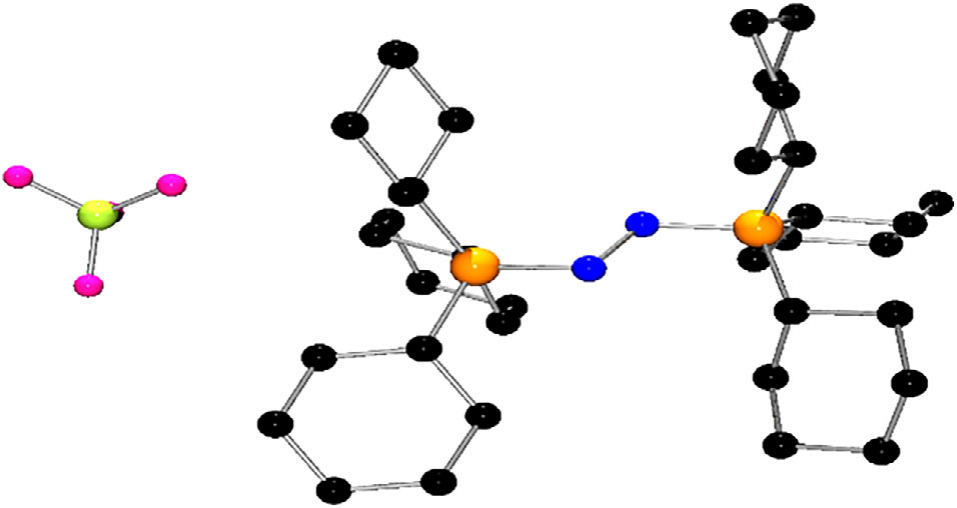
POV‐ray depictions of **5b**. Hydrogen atoms are omitted for clarity. C: black, N: blue, P: orange, B: yellow‐green, F: pink.

Similarly, the reaction of **1** with [Cp_2_Fe][BF_4_] gave an immediate color change to olive‐green; however this dissipated with after a few hours at room temperature suggesting the radical was unstable. Nonetheless, the EPR spectral parameters^[^ ^21^ ^]^ derived from simulation were found to be: *g* = 1.999, *A*_N_ = 19, and *A*_P_ = 49 G) (Figure 5b) inferring the formation of the radical cation salt, [Ph_3_PNNPPh_3_]^•+^[BF_4_]^−^ **6** (Scheme 3). The transient stability of **6** is attributed to the lesser steric demands and poorer electron donor properties of PPh_3_ compared to those in **5b**.

To probe the nature of the radical cations in **5** and **6**, density functional theory (DFT) computations were performed at the PW6B95‐D3/def2‐QZVP+COSMO‐RS // TPSS‐D3/def2‐TZVP+COSMO level of theory in CH_2_Cl_2_ solution.^[^ ^25^, ^26^, ^27^, ^28^, ^29^, ^30^, ^31^, ^32^, ^33^, ^34^, ^35^, ^36^, ^37^, ^38^, ^39^ ^]^ As shown in Figure 7, the respective Wiberg bond indices of about 1.5 and 0.7 for P═N and N─N bonds support the description of the complexes **1** and **2** as the Lewis structures R_3_P═N─N═PR_3_ (R═Ph, Cy), respectively. The respective formation of complexes **1** and **2** from N_2_ and R_3_P (R═Ph, Cy) is highly endergonic by 64.0 and by 61.0 kcal mol^−1^, respectively, and thus thermodynamically forbidden. Oxidation with [Cp_2_Fe][BF_4_] to the radical cations **6^.+^** and **5^.+^** are exergonic by −9.1 and −20.2 kcal mol^−1^, respectively. The respective singly occupied molecular orbital (SOMO) of **6^.+^** and **5^.+^** is primarily the π* orbital perpendicular to the P═N─N═P plane, which is bonding and anti‐bonding with respect to the P═N and N─N bonds, respectively (Figure 7). This is consistent with the observed elongation of the P═N and shortening of the N─N bond‐lengths as seen in the radical cations **6^.+^** and **5^.+^** and further in the dications **6^++^** and **5^++^**. The computed first ionization potential of **1** (101.3 kcal mol^−1^) is higher than that for **2** (89.2 kcal mol^−1^), consistent with more electron poor nature of **1**. Further oxidation of the radical cations to **6^++^** and **5^++^** are predicted to be exergonic by a further −4.2 and −11.4 kcal mol^−1^, respectively, and thus, in principle, thermodynamically accessible. Nonetheless, efforts to generate or observe such doubly oxidized products were unsuccessful, suggesting these species are kinetically unstable. This view is consistent with the observation of irreversible second oxidation waves in the CV of both **1** and **2** (see Supporting Information).

**Figure 7:**
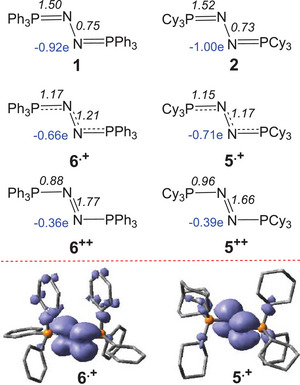
DFT computed NPA charges (in blue) and Wiberg bond indices (top) and spin densities (at isovalue = 0.001) of radical cations **5^.+^** and **6^.+^** (bottom).

In conclusion, we have shown that the *bis*‐phosphazine species R_3_PNNPR_3_ exhibit several forms of reactivity in the presence of boron Lewis acids. These combinations can generate Lewis acid–base adducts, FLPs, or FRPs depending on the precursors. Interestingly, these Lewis acid–base adducts and FLP systems are shown to facilitate the loss of N_2_ from the *bis*‐phosphazine under mild conditions. On the other hand, oxidation of these *bis*‐phosphazines affords the corresponding radical cations, although it appears that the stability of such radical cations is favored by strongly donating and sterically encumbered donors. We are continuing to study these and other main group species incorporating N_2_ fragments.

## Conflict of Interests

The authors declare no conflict of interest.

## Supporting information



Supporting Information

Supporting Information

## Data Availability

The data that support the findings of this study are available in the supplementary material of this article.
