# Supplementary material for: Reactions of R3PNNPR3 Species with Boranes: Classical Adducts, FLPs, and Radical Cations
Source: Angew Chem Int Ed Engl. 2025 Mar 18;64(21):e202503331. doi: 10.1002/anie.202503331 (PMC12087838; doi:10.1002/anie.202503331)
Supplement: Supplementary file 1 — Supporting Information [file ANIE-64-e202503331-s002.pdf]

Supplementary Materials for

Reactions of R_3PNNPR_3 species with Boranes: Classical Adducts, FLPs and Radical Cations

Vaibhav Bedi[a], Andrew Niles L. Ocampochua[a], Zheng-wang Qu[b], Stefan Grimme[b], Douglas W. Stephan*[a]

[a] Department of Chemistry, University of Toronto, Toronto, 80 St. George Street, Ontario M5S 3H6, Canada,

b Mulliken Center for Theoretical Chemistry, Clausius Institut für Physikalische und Theoretische Chemie, Rheinische Friedrich-Wilhelms-Universität Bonn, Beringstrasse 4, 53115 Bonn, Germany, e-mail: qu@thch.uni-bonn.de

Contents

Materials and Methods 2
Preparation of $Cy_3PNNPCy_3$ 2 2
Figure S1. ^1H NMR (400 MHz, C_6D_6, 298 K) spectrum of 2. 3
Figure S2. $^{13}C\{^1H\}$ NMR (126 MHz, C_6D_6, 298 K) spectrum of 2. 4
Figure S3. ^{13}C 135-DEPT NMR (126 MHz, C_6D_6, 298 K) spectrum of 2. 4
Figure S4. $^{31}P\{^1H\}$ NMR (162 MHz, C_6D_6, 298K) spectrum of 2. 5
Figure S5. Mass spectra spectrum of 2. 5
Synthesis of $Ph_3PNN(B(C_6F_5)_3)PPh_3$ 3 6
Figure S6. ^1H NMR (400 MHz, CD_3CN, 298 K) spectrum of 3. 7
Figure S7. $^{13}C\{^1H\}$ NMR (126 MHz, CD_3CN, 298 K) spectrum of 3. 7
Figure S8. ^{11}B NMR (126 MHz, CD_3CN, 298 K) spectrum of 3. 8
Figure S9. $^{19}F\{^1H\}$ NMR (162 MHz, CD_3CN, 298K) spectrum of 3. 8
Figure S10. Mass Spectrometry Data of $Ph_3PNN(B(C_6F_5)_3)PPh_3$ 3 9
Synthesis of $Ph_3PN(BF_3)N(BF_3)PPh_3$ 4 9
Figure S11. ^1H NMR (400 MHz, CD_3CN, 298 K) spectrum of 4. 10
Figure S12. $^{13}C\{^1H\}$ NMR (126 MHz, CD_3CN, 298 K) spectrum of 4. 10
Figure S13. $^{31}P\{^1H\}$ NMR (162 MHz, CD_3CN, 298K) spectrum of 4. 11
Figure S14. ^{11}B NMR (126 MHz, CD_3CN, 298 K) spectrum of 4. 11
Figure S15. $^{19}F\{^1H\}$ NMR (162 MHz, CD_3CN, 298K) spectrum of 4 11
Thermolysis of 3 12
Figure S16. $^{31}P\{^1H\}$ NMR (162 MHz, ODFB, 298K) spectrum of thermolysis of 3. 12
Figure S17. ^{11}B NMR 128 MHz, ODFB, 298K) spectrum of thermolysis of 3. 13
Figure S18. $^{19}F\{^1H\}$ NMR (377 MHz, ODFB, 298K) spectrum of thermolysis of 3. 13
Thermolysis of 1 in the presence of BPh_3 13
Figure S19. $^{31}P\{^1H\}$ NMR spectrum of thermolysis of 1/BPh_3. 14
Figure S20. ^{11}B NMR spectrum of thermolysis of 1/BPh_3. 14
Synthesis of $[Cy_3PNNPCy_3]^{\bullet+}$ $^{-\bullet}[B(C_6F_5)_3]$ 5a 14
Figure S24. EPR spectrum of 5a, experimental black; simulation green. 15
Synthesis of $[Cy_3PNNPCy_3]^{\bullet+}$ $[BF_4]$ 5b 15
Figure S25. EPR spectrum of 5b, experimental black; simulation green. 16
UV-Vis Spectra of $[Cy_3PNNPCy_3]^{\bullet+}$ $[BF_4]$ 5b 16
Generation of $[Ph_3PNNPPh_3]^{\bullet+}$ $[BF_4]$ 6 17
Figure S26. EPR spectrum of 6, experimental black; simulation green. 17
CV Studies 18
data for $[Cy_3PNNPCy_3]$ 18

data for [$Ph_3PNNPPh_3$] 19
Computational Details 20
References 34

Materials and Methods

General considerations: All manipulations were performed in a Glove box MB LABmaster produced by MBraun or using standard Schlenk techniques under an inert atmosphere of anhydrous N_2 unless otherwise mentioned. All glassware and Teflon-coated stir bars were oven-dried and cooled under vacuum before use. Dry, oxygen-free pentane, toluene and dichlormethane was prepared using an Innovative Technologies solvent purification system and stored over activated 4 Å molecular sieves before use. Tetrahydrofuran was distilled from sodium-benzophenone ketyl and stored over activated 4 Å molecular sieves before use. Fluorobenzene and 1,2-difluorobenzene were distilled over calcium hydride and stored over activated 4 Å molecular sieves before use. Deuterated dichloromethane (CD_2Cl_2), chlorform ($CDCl_3$), benzene (C_6D_6), tetrahydrofuran (C_4D_8O), and toluene (C_7D_8) were purchased from Sigma-Aldrich, distilled from calcium hydride or sodium-benzophenone ketyl, and stored over molecular sieves (4 Å) for at least two days prior to use. Commercial reagents were purchased from Sigma-Aldrich, Strem Chemicals, TCI Chemicals or Alfa Aesar, and were used without further purification unless indicated otherwise. Compound $Ph_3PNNPPh_3$ **1** was prepared by literature methods.[1]
NMR spectra were obtained on a Bruker AvanceIII-400 MHz spectrometer or an Agilent DD2 500 MHz spectrometer. ^{1}H NMR data are reported relative to protio-solvent signals as follows: chemical shift (δ/ppm), coupling constant (Hz), normalized integrals. $^{13}C\{^{1}H\}$ NMR chemical shifts (δ/ppm), are referenced relative to protio-solvent signals. $^{31}P\{^{1}H\}$, ^{19}F and ^{11}B NMR chemical shifts (δ/ppm) are reported relative to H_3PO_4, $CFCl_3$ and $BF_3 \cdot OEt_2$ external standards. Bulk analysis by elemental analysis or mass spectroscopy for some compounds could not be carried out due to compound degradation or decomposition. X-ray data were collected on a Bruker Apex II diffractometer at 150(±2) K for all crystals. All EPR spectra were acquired using a Bruker CW X-band ECS-EMXplus EPR Spectrometer (9.34 GHz) equipped with a HS4119VT EPR cavity. UV/Vis spectra was recorded on Lambda 365 UV/Vis Spectrometer: Spectral range of 190 nm to 1100 nm (Double beam instrument).

Preparation of $Cy_3PNNPCy_3$ 2

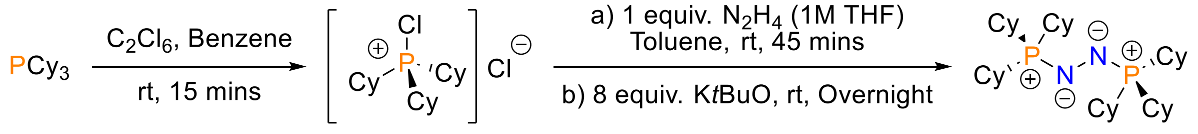

95% **2, 40%**

Synthesis of [Cy_3PCl]Cl - The published synthetic protocol was followed.[2] ^{31}P NMR ($CDCl_3$): δ = 107.6 (s) in accord with that reported by Jolly *et. al.*[3] ^{31}P NMR (162 MHz, CD_2Cl_2) δ = 107.2.

Synthesis of 2 - To a 20 mL scintillation vial containing a solution of [Cy_3PCl]Cl (1.00 g, 2.85 mmol, 1 equiv.) in 10 mL Toluene was added dropwise via syringe N_2H_4 (1 M in THF, 2.85 mL, 2.85 mmol, 1 equiv.) at room temperature. The reaction mixture was stirred for 45 minutes and then K*t*BuO (8 equiv.) was added to the slurry. After stirring the reaction mixture overnight, it was filtered through dried silica. The resultant filtrate was concentrated to approximately 2 mL and triturated with 6 mL of diethyl ether x2, decanted, and evacuated to afford yellowish-

white powder. It was further purified by recrystallizing it with ODFB:Hexane (1:5) at -35 °C. The supernatant was decanted and the remaining solid was dried in vacuo to afford bright yellow crystals of $Cy_3PNNPCy_3$ 2 (0.67 g, 1.13 mmol, 40% yield).

^{1}H NMR (400 MHz, C_6D_6, 298 K): δ 2.32-2.30 (m, 6H, *ipso*-CH), 2.20-2.16 (m, 12H, *p*-CH_2), 1.85-1.80 (m, 24H, *o*-CH_2), 1.28-1.25 (m, 24H, *m*-CH_2)
^{13}C{^{1}H} NMR (126 MHz, C_6D_6, 298 K): δ 34.32, 34.06, 33.80 (t, p-CH_2), 28.12-27.99 (m, o,m-CH_2), 27.20 (s, *ipso*-CH)
^{13}C 135-DEPT NMR (126 MHz, C_6D_6, 298 K): δ 34.32, 34.06, 33.80 (t, p-CH_2), 28.12-27.99 (m, o,m-CH_2), 27.20 (s, *ipso*-CH)
^{31}P{^{1}H} NMR (162 MHz, C_6D_6, 298K): δ 27.2 (s)

Figure S1. ^{1}H NMR (400 MHz, C_6D_6, 298 K) spectrum of 2.

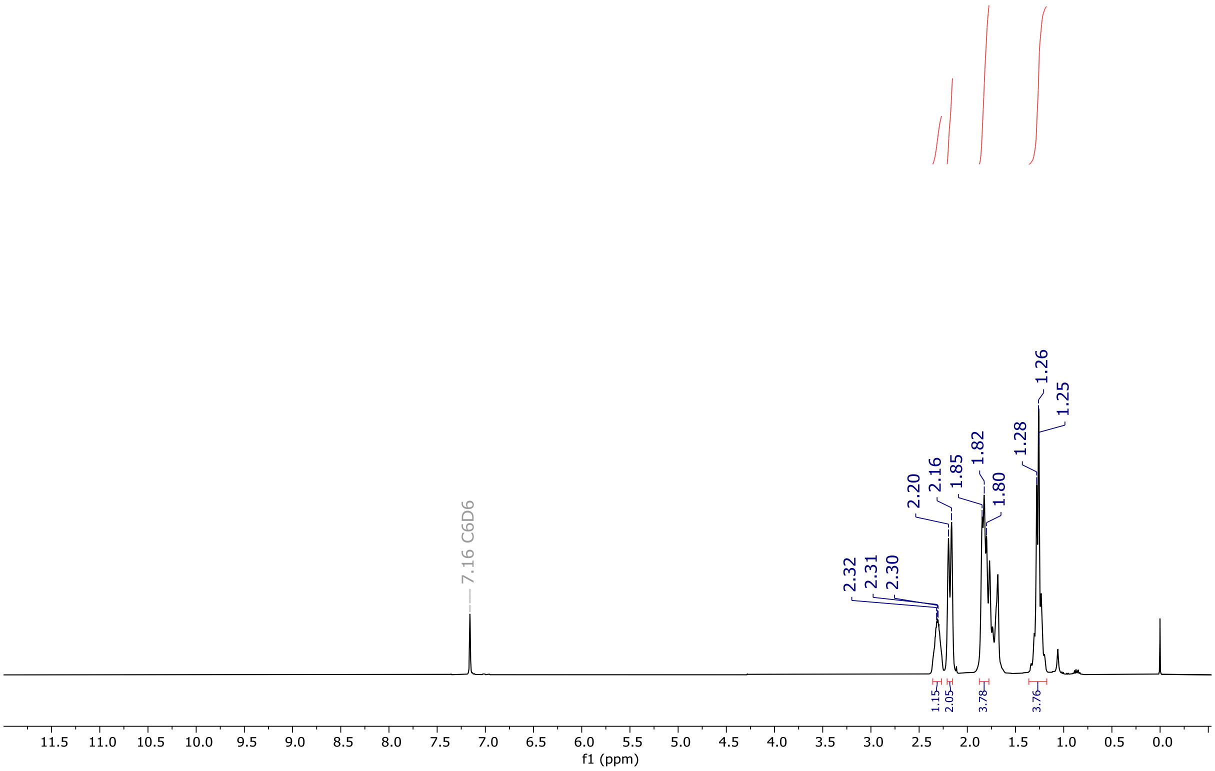

Figure S2. $^{13}C\{^1H\}$ **NMR (126 MHz,** C_6D_6**, 298 K) spectrum of 2.**

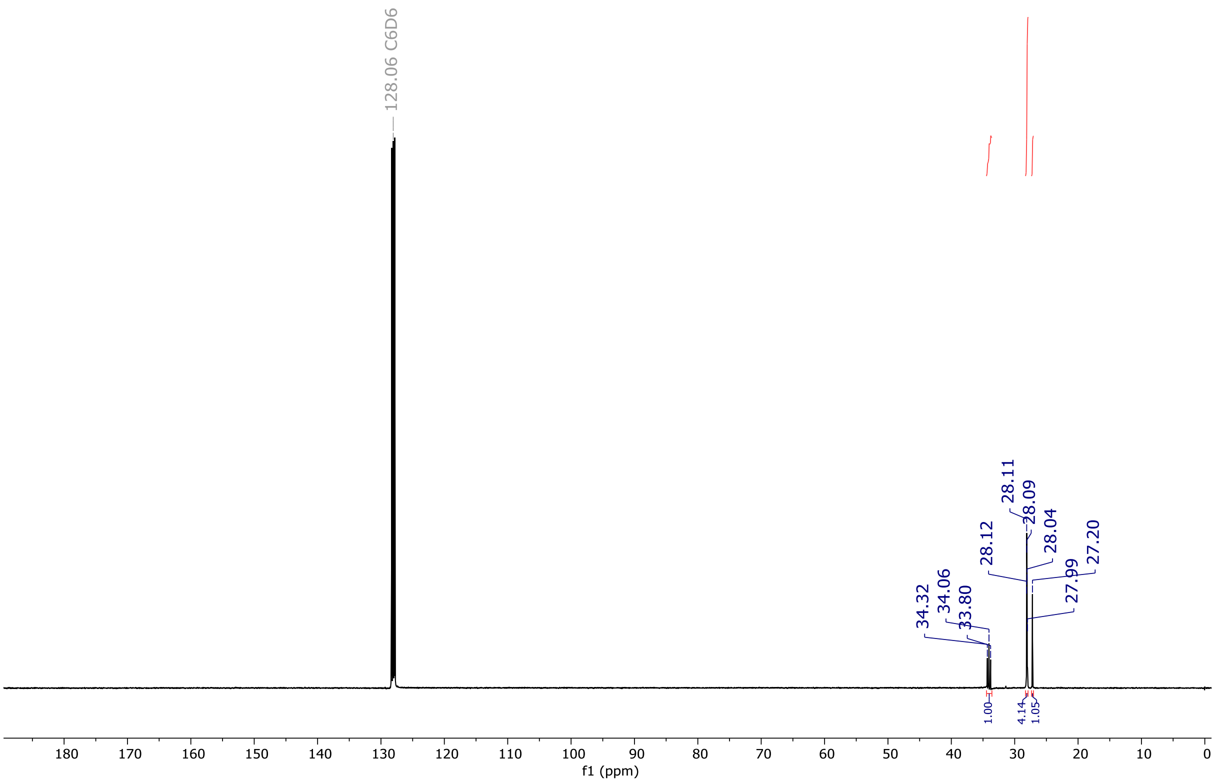

Figure S3. ^{13}C **135-DEPT NMR (126 MHz,** C_6D_6**, 298 K) spectrum of 2.**

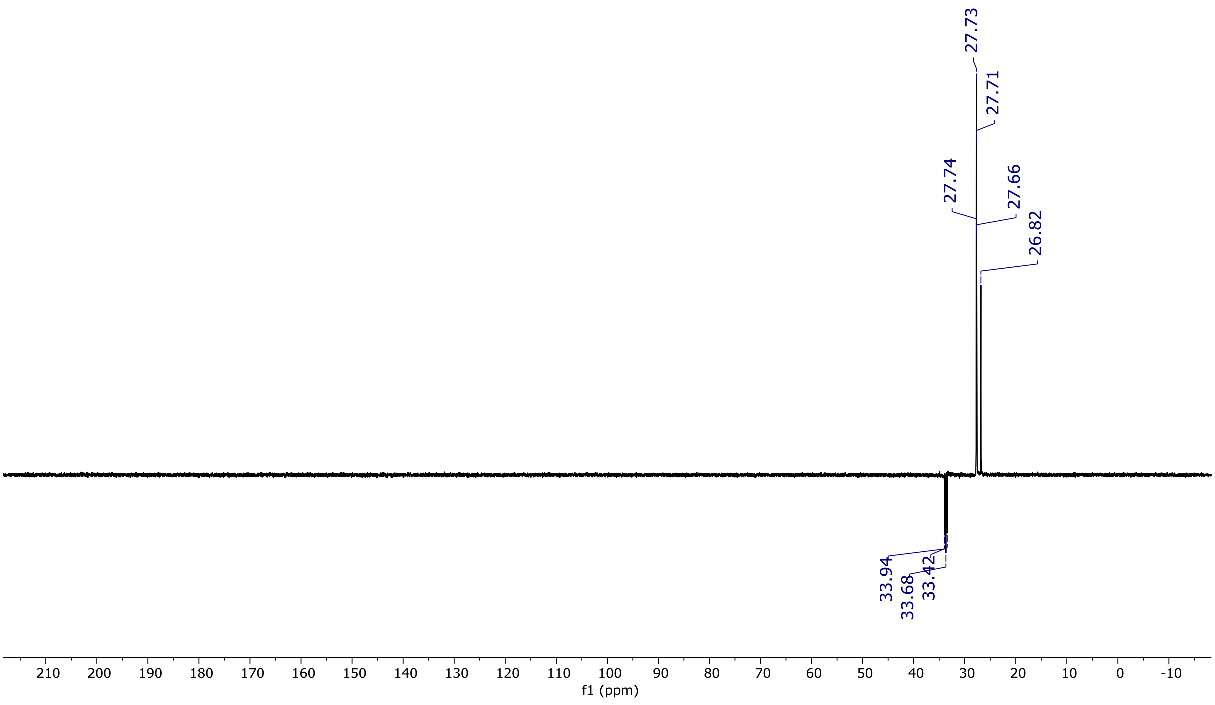

Figure S4. $^{31}P\{^1H\}$ NMR (162 MHz, C_6D_6, 298K) spectrum of 2.

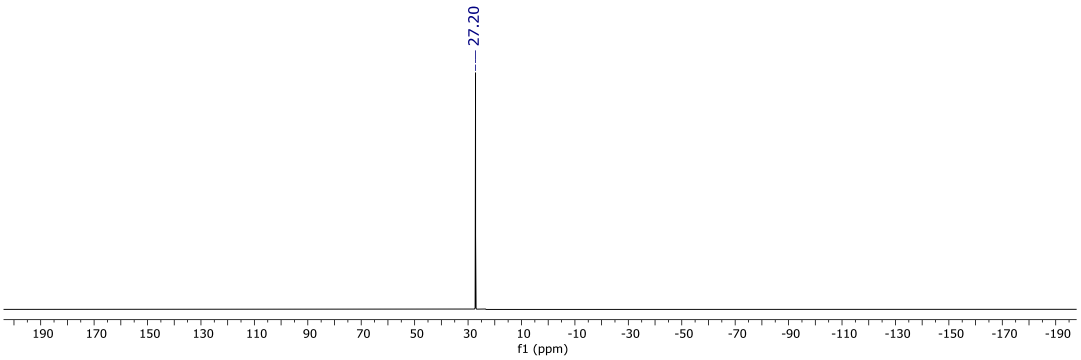

Figure S5. Mass spectra spectrum of 2.

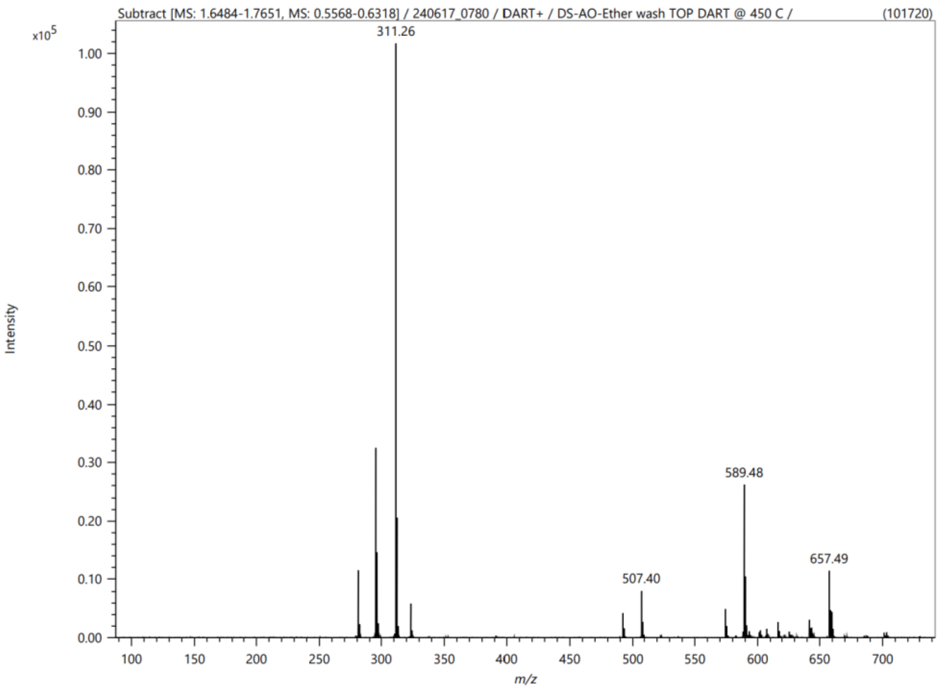

Spectrum

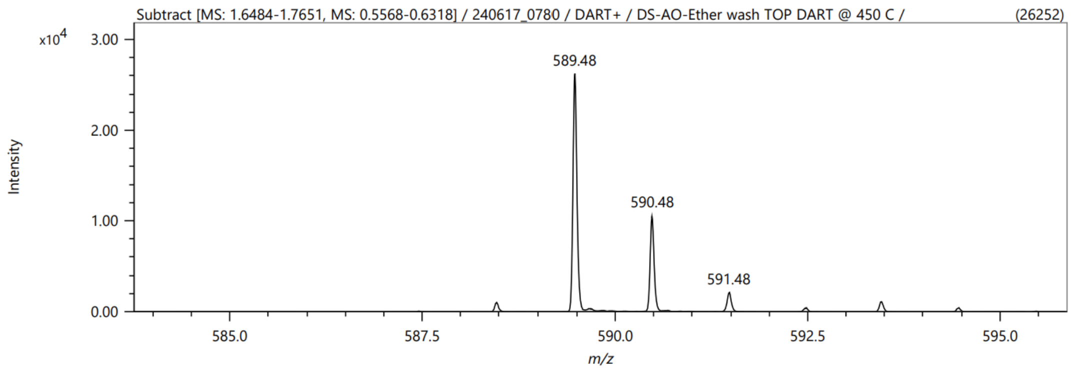

Elemental Composition

Parameters		Elements Set 1:					
		Symbol	C	H	O	N	P
Tolerance:	±5.00 mDa	Min	0	0	0	0	0
Electron:	Even	Max	100	200	20	10	2
Charge:	+1						
DBE:	-1.5 - 100.0						

Results

Mass	Intensity	Formula	Calculated Mass	Mass Difference [mDa]	Mass Difference [ppm]	DBE
589.47706	26252.16	C44 H61	589.47678	0.28	0.48	14.5
		C36 H67 N2 P2	589.47740	-0.34	-0.57	5.5
		C28 H61 N8 O5	589.47594	1.12	1.90	2.5
		C32 H65 N2 O7	589.47863	-1.57	-2.66	1.5
		C27 H62 N10 O2 P	589.47893	-1.87	-3.18	2.5
		C37 H66 O3 P	589.47441	2.65	4.50	5.5

1 / 2

Mass	Intensity	Formula	Calculated Mass	Mass Difference [mDa]	Mass Difference [ppm]	DBE
		C33 H61 N6 O3	589.47997	-2.90	-4.93	6.5
		C31 H67 N4 O2 P2	589.47338	3.68	6.25	1.5
		C39 H61 N2 O2	589.47276	4.31	7.30	10.5
		C31 H66 N4 O4 P	589.48162	-4.56	-7.73	1.5

Synthesis of $Ph_3PNN(B(C_6F_5)_3)PPh_3$ 3

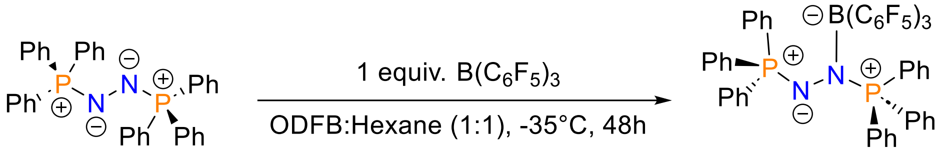

1 **3, 68%**

In a 4 mL vial, a 1 mL solution of 1 (10 mg, 0.018 mmol) in ODFB was prepared and layered with a 1 ml 1 equivalent solution of $B(C_6F_5)_3$ (9 mg, 0.018 mmol) in hexane at -35°C. This afforded white crystals of $Ph_3PNN(B(C_6F_5)_3)PPh_3$ in 68% (13 mg) yield in 48h after decanting the mother liquor, washing with cold hexane, and evacuating the solvent.

^{1}H NMR (400 MHz, CD_3CN) δ 7.63-7.29 (*m*, Ar-H, $Ph_3PNN(B(C_6F_5)_3)PPh_3$)

^{13}C{^{1}H} NMR (101 MHz, CD_3CN) δ 138.35-129.65 (*m*, Ar-H)

^{31}P{^{1}H} NMR (162 MHz, CD_3CN) δ 46.7 (s, 1P), 45.5 (s)

^{11}B NMR (128 MHz, CD_3CN): δ -4.4 (s)

^{19}F{^{1}H} NMR (377 MHz, CD_3CN): δ -134.97 (2F, *o*-F), -161.40 (4F, *p*-F), -165.60 (4F, *m*-F)

Figure S6. ^{1}H NMR (400 MHz, CD3CN, 298 K) spectrum of 3.

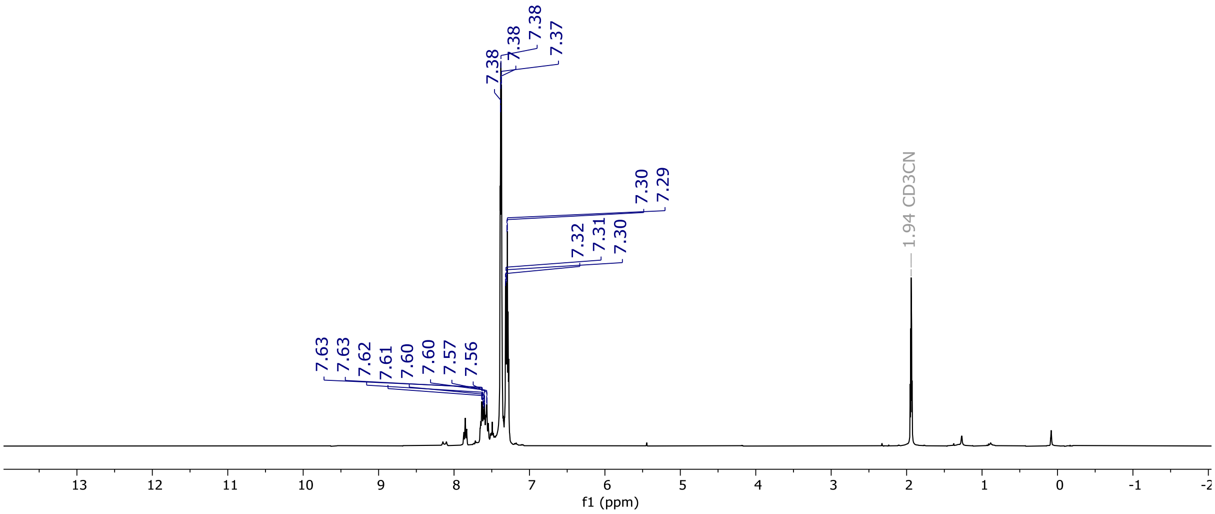

Figure S7. ^{13}C{^{1}H} NMR (126 MHz, CD3CN, 298 K) spectrum of 3.

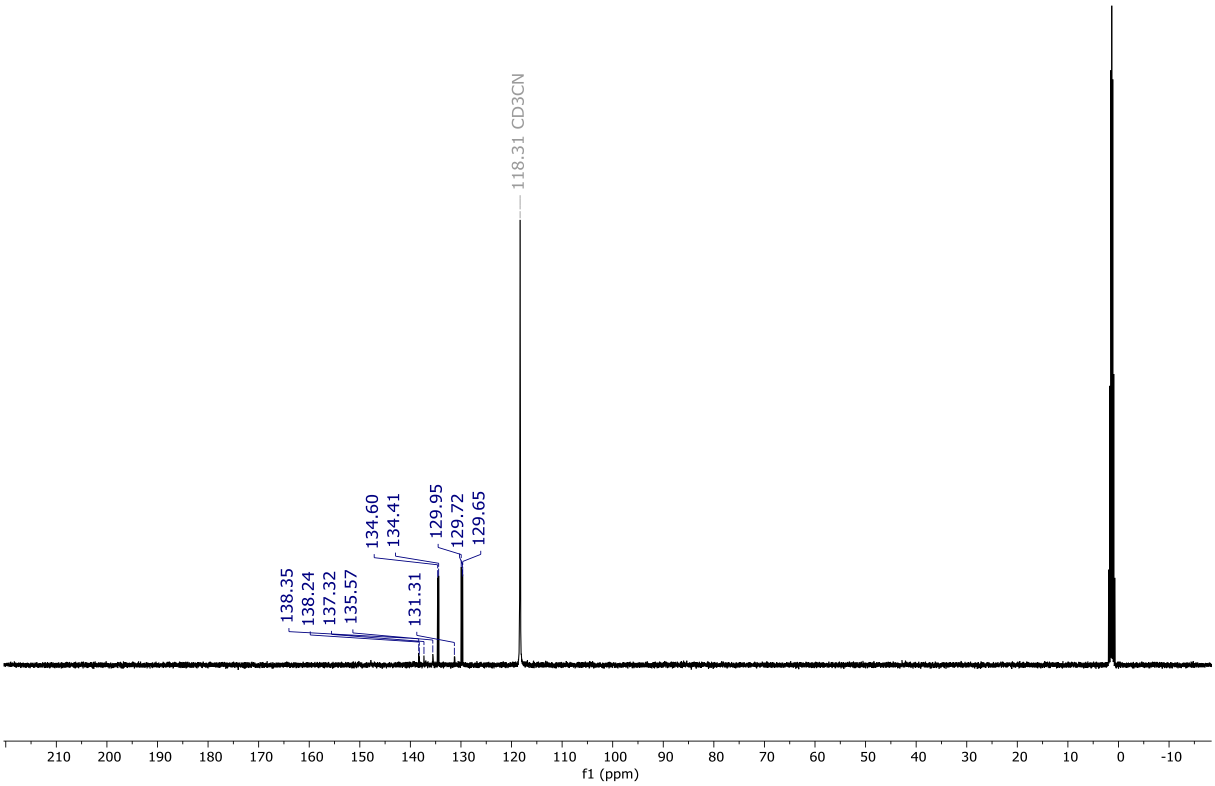

Figure S8. $^{31}P\{^{1}H\}$ NMR (162 MHz, CD_3CN, 298K) spectrum of 3.

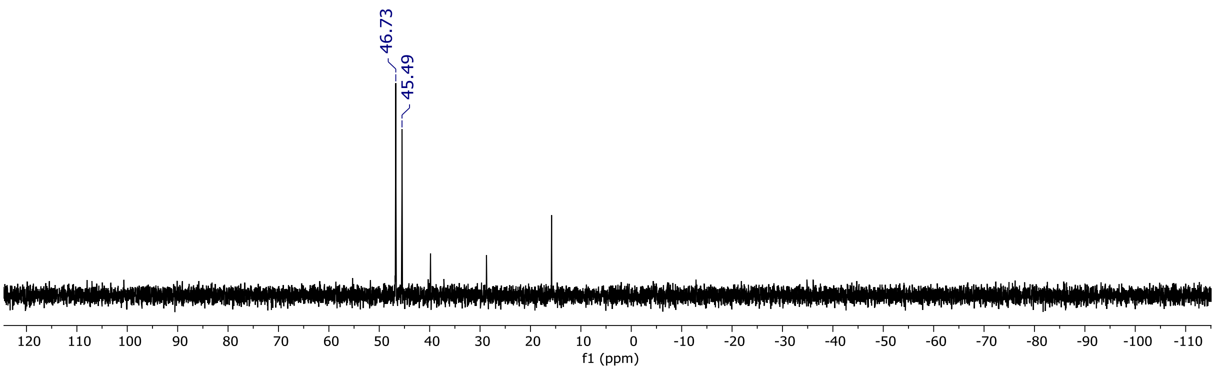

Figure S8. ^{11}B NMR (126 MHz, CD_3CN, 298 K) spectrum of 3.

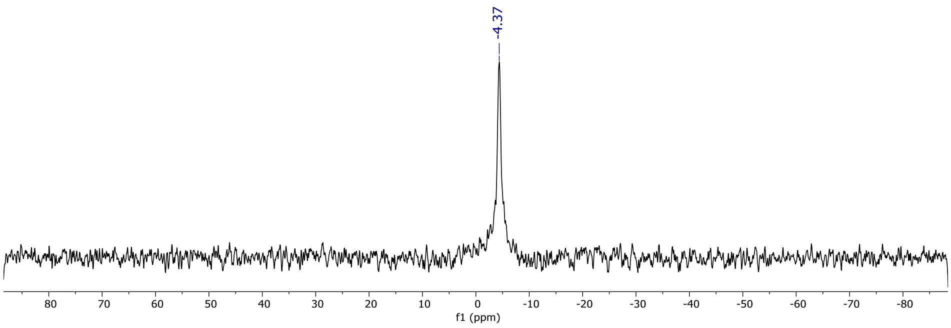

Figure S9. $^{19}F\{^{1}H\}$ NMR (162 MHz, CD_3CN, 298K) spectrum of 3.

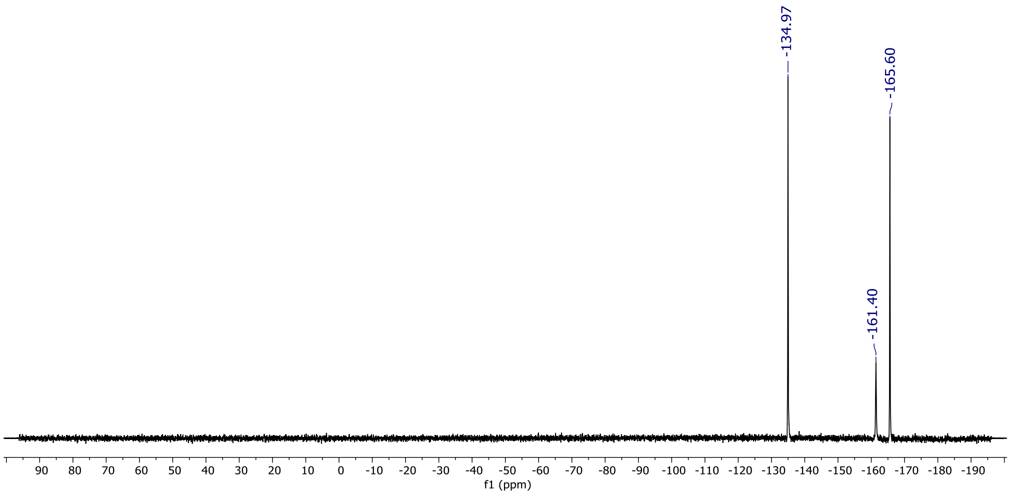

Figure S10. Mass Spectrometry Data of $Ph_3PNN(B(C_6F_5)_3)PPh_3$ 3

Theoretical - 1064.17 m/z, Experimental – 1064.07 m/z

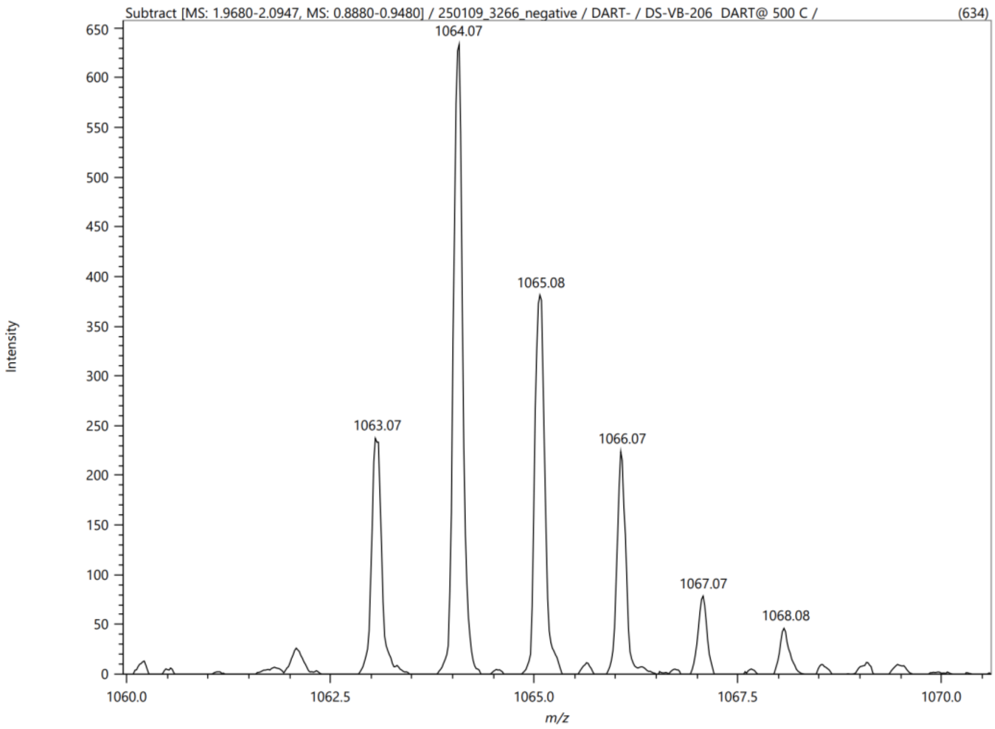

Synthesis of $Ph_3PN(BF_3)N(BF_3)PPh_3$ 4

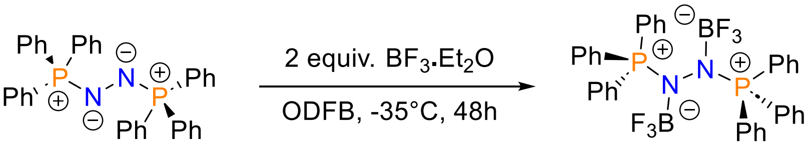

1 4, 82%

In a 4 mL vial, 1 mL solution of 1 (10 mg, 0.018 mmol) in ODFB was prepared and layered with 2 equivalents of $BF_3.OEt_2$ (5 mg, 0.036 mmol, 4.6 µL) at -35 °C, which afforded white crystals of $Ph_3PN(BF_3)N(BF_3)PPh_3$ in 82% (10 mg) yield after decanting the mother liquor and washing with cold hexane.

^{1}H NMR (400 MHz, CD_3CN) δ 7.88, 7.87, 7.85 (t, *p*-Ar-H), 7.65-7.59 (m, *o,m*-Ar-H)

^{13}C{^{1}H} NMR (126 MHz, CD_3CN) δ 137.21-131.17 (*m*, Ar-C)

^{31}P{^{1}H} NMR (162 MHz, CD_3CN) δ 46.4 (*s*)

^{11}B NMR (128 MHz, CD_3CN) δ -0.2 (*s*)

^{19}F{^{1}H} NMR (377 MHz, CD_3CN) δ -151.85 (*s*)

Figure S11. ^{1}H NMR (400 MHz, CD3CN, 298 K) spectrum of 4.

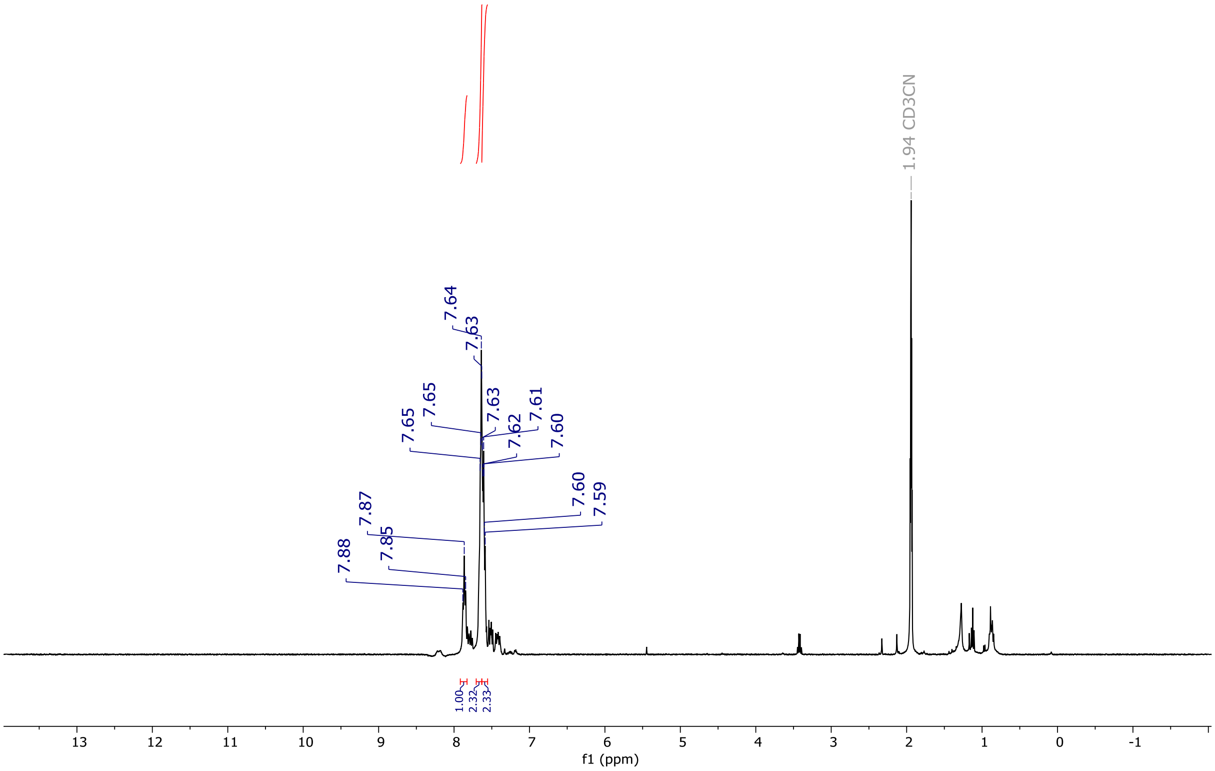

Figure S12. ^{13}C{^{1}H} NMR (126 MHz, CD3CN, 298 K) spectrum of 4.

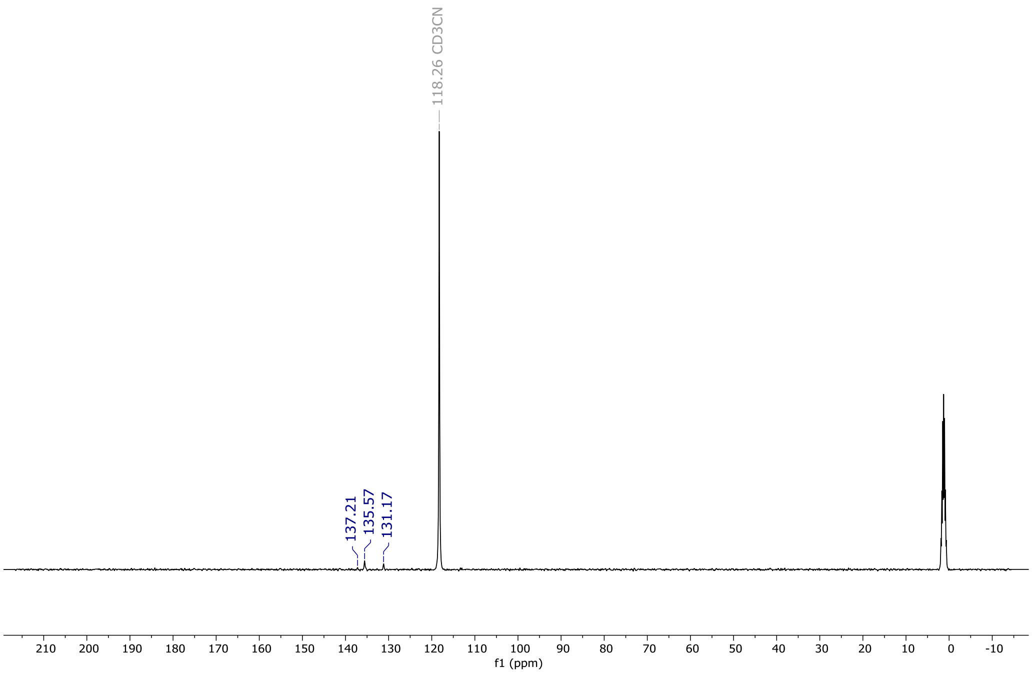

Figure S13. $^{31}P\{^1H\}$ **NMR (162 MHz, CD_3CN, 298K) spectrum of 4.**

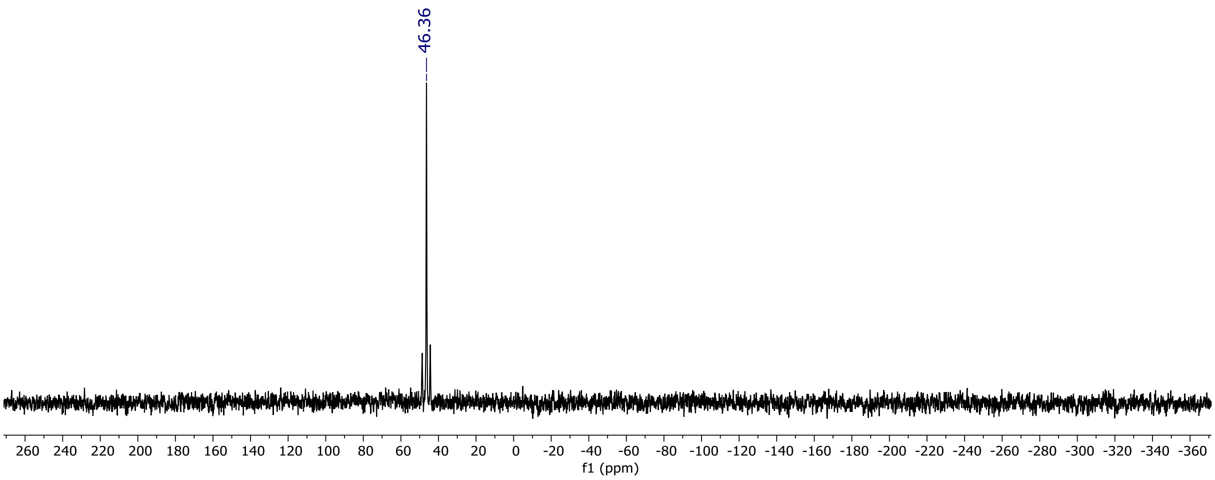

Figure S14. ^{11}B **NMR (126 MHz, CD_3CN, 298 K) spectrum of 4.**

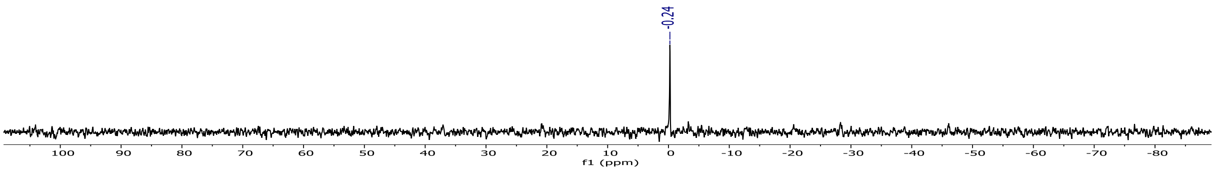

Figure S15. $^{19}F\{^1H\}$ **NMR (162 MHz, CD_3CN, 298K) spectrum of 4**

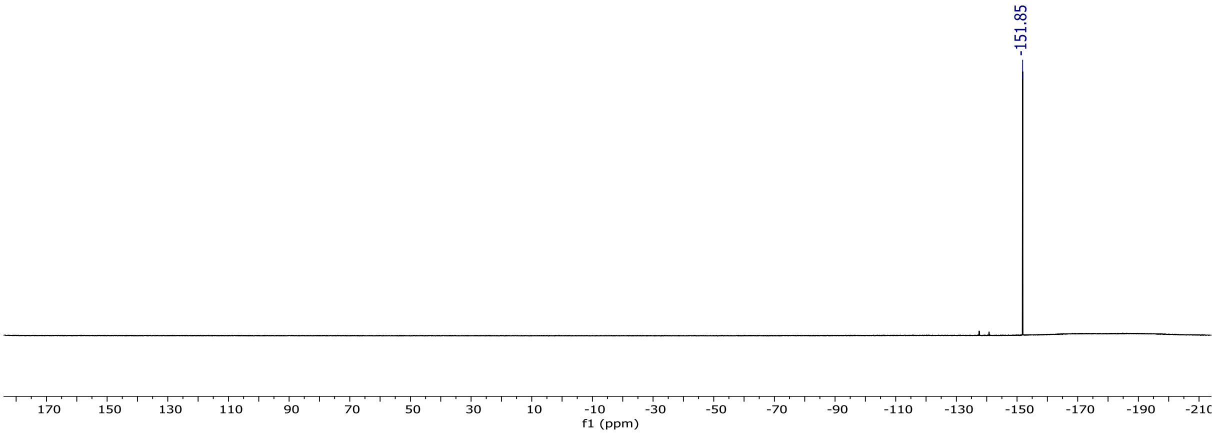

Thermolysis of 3

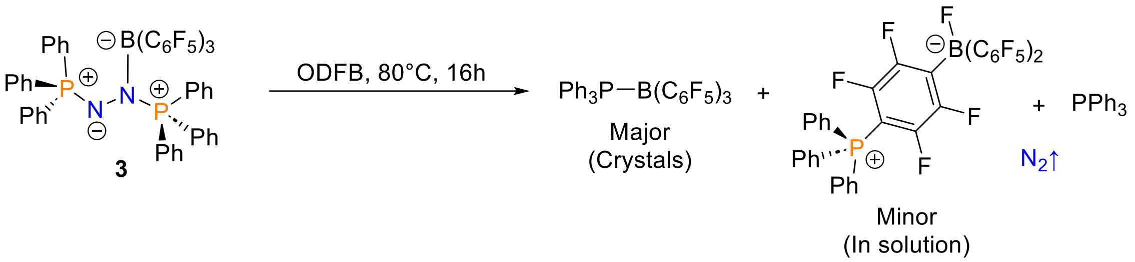

Compound **3** (10 mg, 0.0094 mmol) was taken up in 0.7 mL of ODFB and transferred to a J-Young tube. The yellowish-white solution was heated to 80 °C for 12 hours. After the solution was cooled to room temperature, white crystals crashed out of the solution. The crystals were isolated and found to be of $Ph_3P\text{-}B(C_6F_5)_3$ (34 mg, 0.004 mmol, 47% yield) based on known X-ray crystallographic data.[4] The remaining mother liquor was monitored by $^{31}P\{^1H\}$, ^{19}F and ^{11}B NMR spectroscopy, indicating the presence of *para*-attacked $Ph_3P(C_6F_4)BF(C_6F_5)_2$ and PPh_3.

$^{31}P\{^1H\}$ NMR (162 MHz, ODFB, 298K) δ 46.7 (s, *β*-P(to $B(C_6F_5)_3$)), 45.5 (s, *γ*-P(to $B(C_6F_5)_3$), 15.8 (s, $P(C_6F_4)BF(C_6F_5)_2$), -4.3 (PPh_3).

^{11}B NMR (128 MHz, ODFB, 298 K): δ -1.0 (br s) , -3.8 (br s)

^{19}F NMR (377 MHz, ODFB, 298K) δ -132.93-(-132.96) (m, $-C_6F_4$, $P(C_6F_4)BF(C_6F_5)_2$), -133.00-(-133.02) (m, $-C_6F_4$, $P(C_6F_4)BF(C_6F_5)_2$), -133.47 (br s, *o*-$B(C_6F_5)_2$, $P(C_6F_4)BF(C_6F_5)_2$), -136.17(s, *o*-F, $Ph_3PNN(B(C_6F_5)_3)PPh_3$), -159.20 (t, 2F, 3 $J_{F\text{-}F}$ = ~19 Hz, para-C_6F_5, $P(C_6F_4)BF(C_6F_5)_2$), -160.39 (s, *m*-F, $Ph_3PNN(B(C_6F_5)_3)PPh_3$), -165.39 (m, m-$C_6F_5$, $P(C_6F_4)BF(C_6F_5)_2$), -165.63(s, *m*-F, $Ph_3PNN(B(C_6F_5)_3)PPh_3$),

Figure S16. $^{31}P\{^1H\}$ NMR (162 MHz, ODFB, 298K) spectrum of thermolysis of 3.

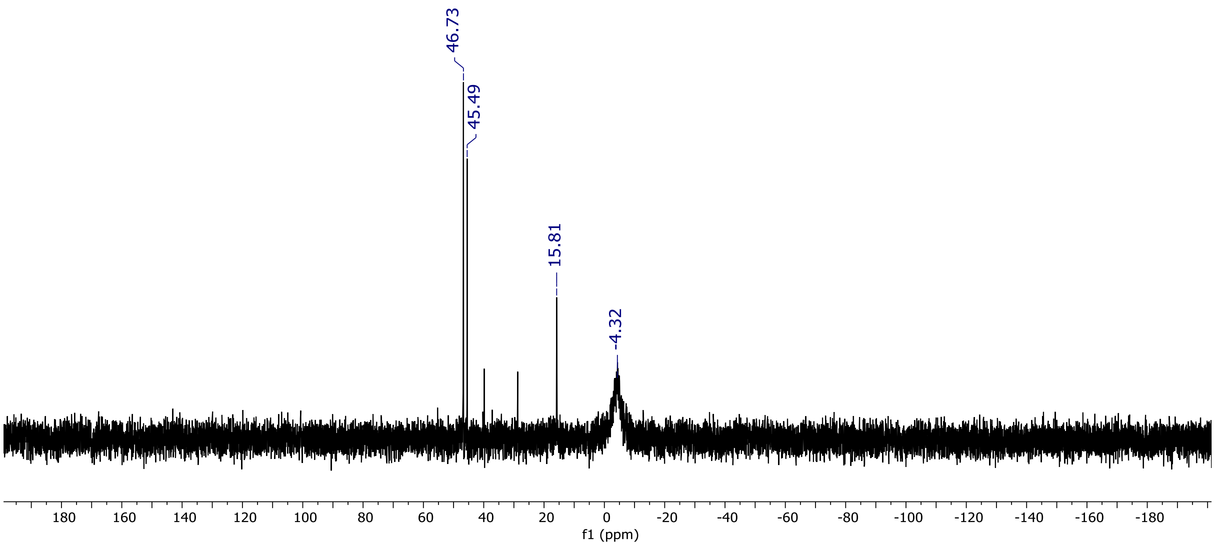

Figure S17. ^{11}B NMR 128 MHz, ODFB, 298K) spectrum of thermolysis of 3.

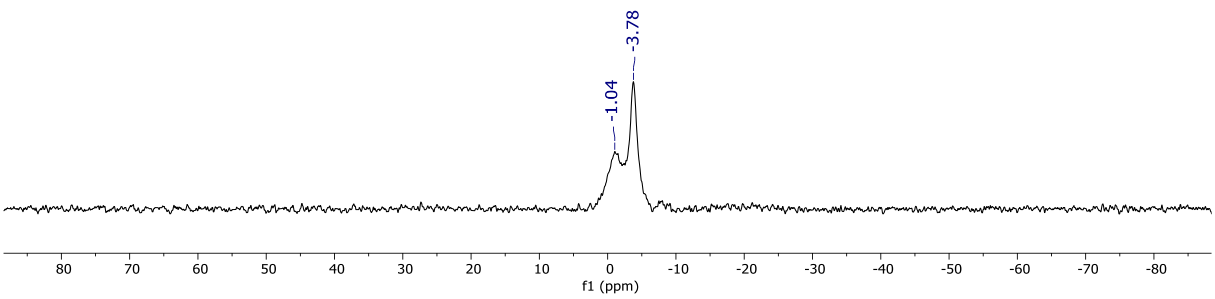

Figure S18. ^{19}F{^{1}H} NMR (377 MHz, ODFB, 298K) spectrum of thermolysis of 3.

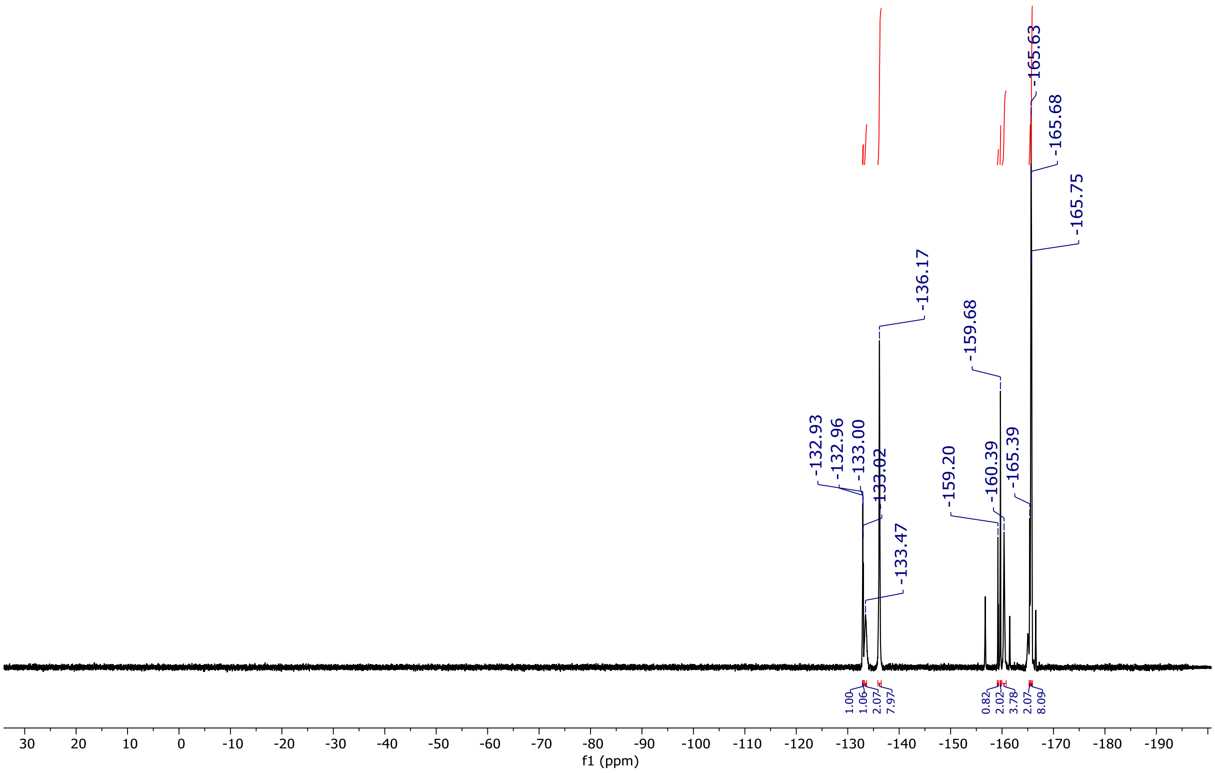

Thermolysis of 1 in the presence of BPh_3

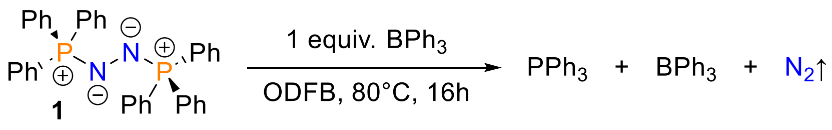

Compound **1** (0.010 g, 0.018 mmol) and 1 equivalent BPh_3 (4.38 mg, 0.018 mmol) was taken up in 0.7 mL of CH_2Cl_2 and transferred to a J-Young tube. The deep red solution was heated to

80 °C for 12 hours and monitored by $^{31}P\{^{1}H\}$ and ^{11}B NMR spectroscopy, indicating the color change to white solution and formation of Ph_3P and BPh_3 remained unreacted.
$^{31}P\{^{1}H\}$ NMR (162 MHz, CH_2Cl_2, 298K): δ -5.3 (s, 2P, PPh_3).
^{11}B NMR (128 MHz, CH_2Cl_2, 298 K): δ 62.3s, 1B, BPh_3)

Figure S19. $^{31}P\{^{1}H\}$ NMR spectrum of thermolysis of 1/BPh_3.

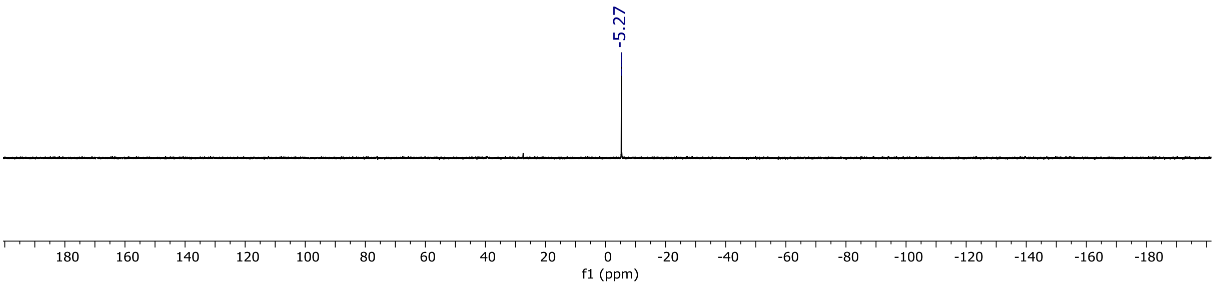

Figure S20. ^{11}B NMR spectrum of thermolysis of 1/BPh_3.

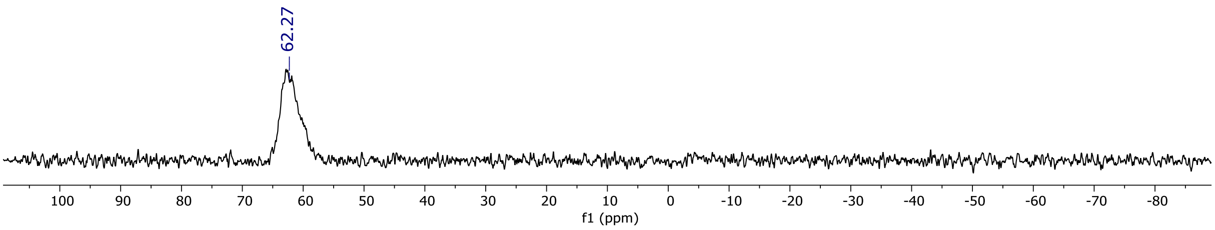

Synthesis of $[Cy_3PNNPCy_3]^{\bullet +}\ ^{-\bullet}[B(C_6F_5)_3]$ 5a

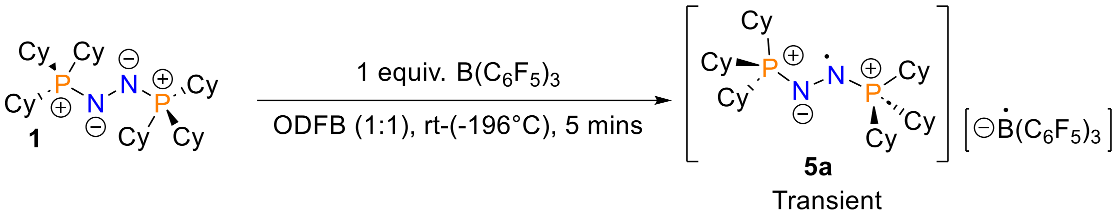

To a 10 mL scintillation vial, a yellow solution of 2 (10 mg, 0.017 mmol) in ODFB was prepared. Another colorless solution of 1 equivalent $B(C_6F_5)_3$ (**9** mg, 0.017 mmol) in ODFB was prepared similarly and added to the 2 solution dropwise while stirring. It resulted in an

instant color change from yellow to dark olive green, which was then transferred to a J-Young Tube and plunged in liquid nitrogen (-196 °C) to stop further reaction. EPR was taken to confirm its presence.
EPR: g = 2.002, A_N = 20 G, A_P = 56 G

Figure S24. EPR spectrum of 5a, experimental black; simulation green.

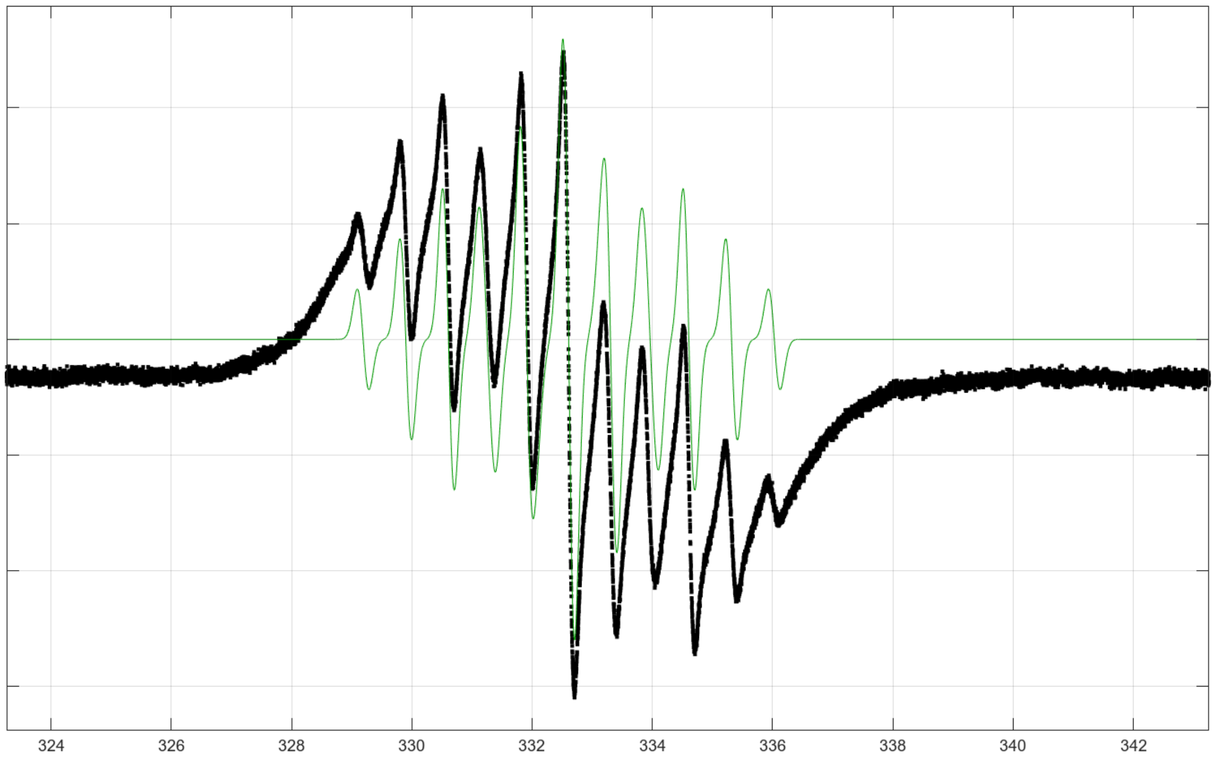

Synthesis of $[Cy_3PNNPCy_3]^{\bullet+}$ $[BF_4]$ 5b

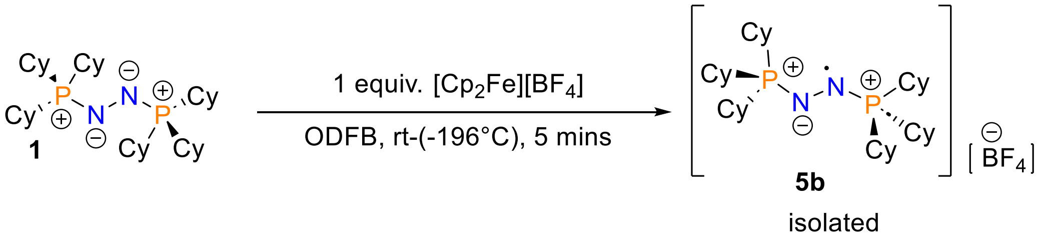

To a 10 mL scintillation vial, a yellow solution of 2 (10 mg, 0.017 mmol) in ODFB was prepared. Another black solution of 1 equivalent $[(Cp)_2Fe][BF_4]$ (4.63 mg, 0.017 mmol) in ODFB was prepared in a similar manner and it was added to the 2 solution dropwise while stirring. It resulted in an instant color change from yellowish-black to dark olive green, which was then transferred to a J-Young Tube and plunged in liquid nitrogen (-196 °C) to stop further reaction. After taking the EPR spectra, the solution was concentrated and greenish blue crystals were isolated with ODFB:Ether (1:1) diffusion method.
EPR: g = 1.999, A_N = 19 G, A_P = 49 G.

Figure S25. EPR spectrum of 5b, experimental black; simulation green.

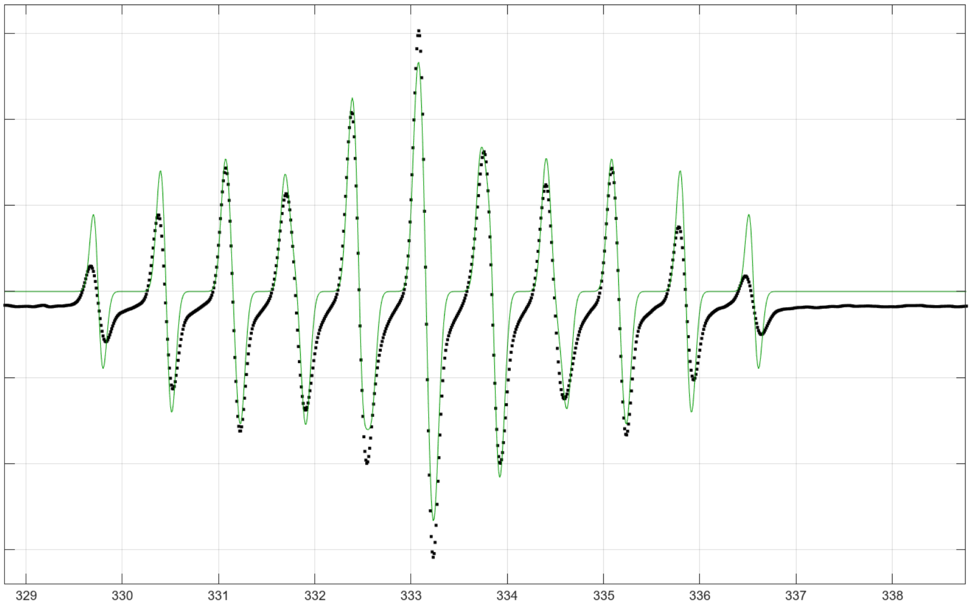

UV-Vis Spectra of $[Cy_3PNNPCy_3]^{\bullet+}$ $[BF_4]$ 5b

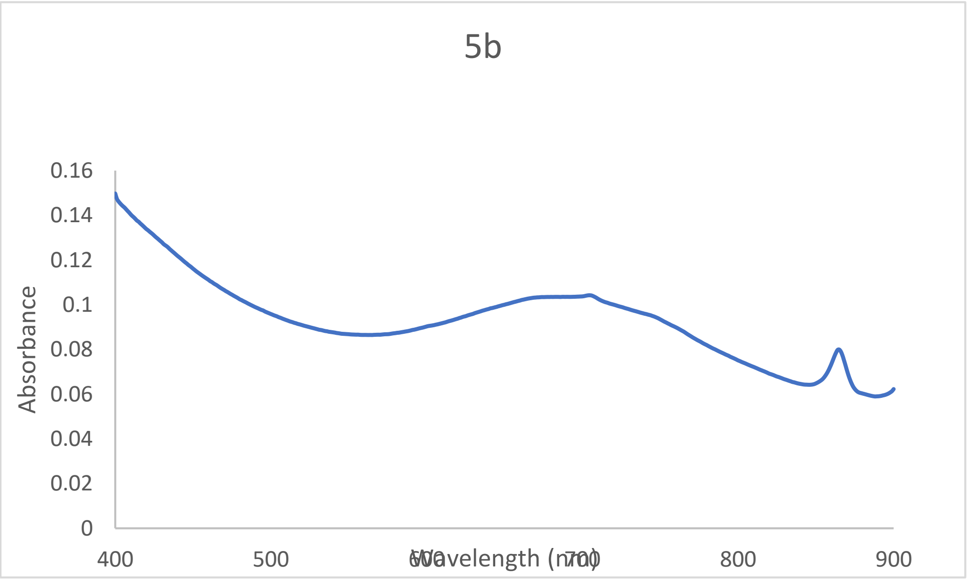

Max Absorbance: 705 nm (ε = 17.5 $M^{-1}cm^{-1}$)

Generation of $[Ph_3PNNPPh_3]^{\bullet+}$ $[BF_4]$ 6

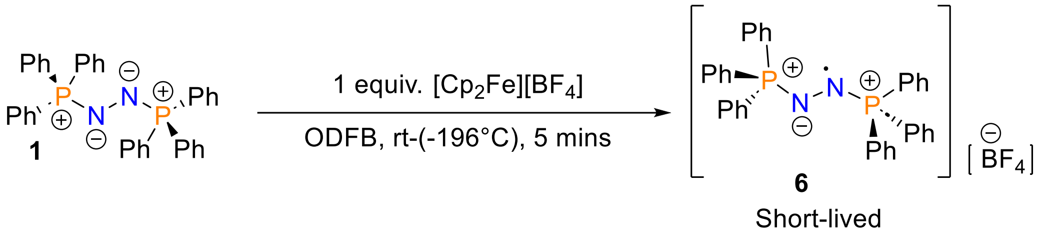

To a 10 mL scintillation vial, a deep red solution of 1 (10 mg, 0.018 mmol) in ODFB was prepared. Another black solution of 1 equivalent $[(Cp)_2Fe][BF_4]$ (4.91 mg, 0.018 mmol) in ODFB was prepared similarly and it was added to the 1 solution dropwise while stirring. It resulted in an instant color change from deep red to bluish-green, which was then transferred to a J-Young Tube and plunged in liquid nitrogen (-196 °C) to stop further reaction.
EPR: g = 1.999, AN = 19 G, AP = 49 G.

Figure S26. EPR spectrum of 6, experimental black; simulation green.

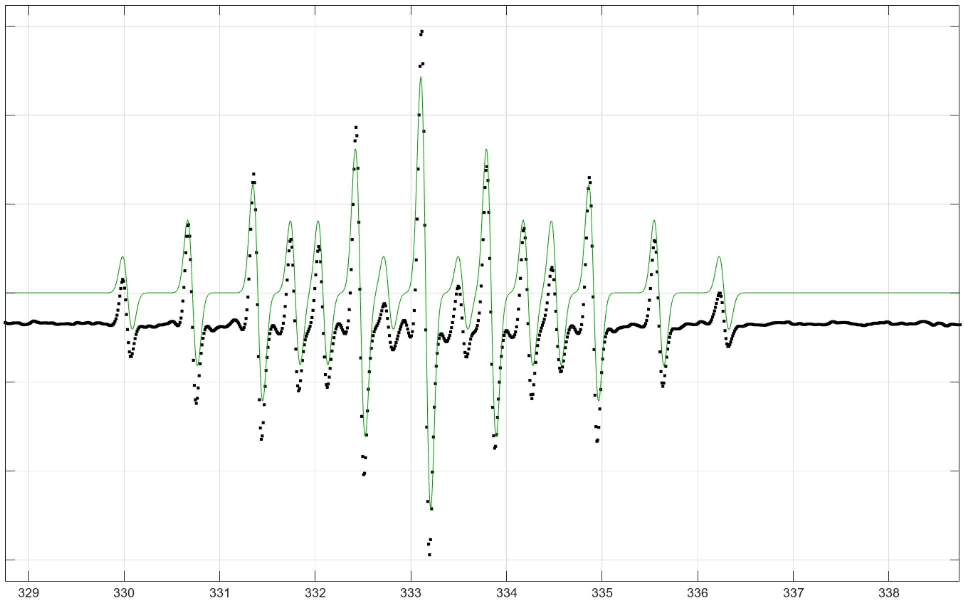

CV Studies

data for [$Cy_3PNNPCy_3$]

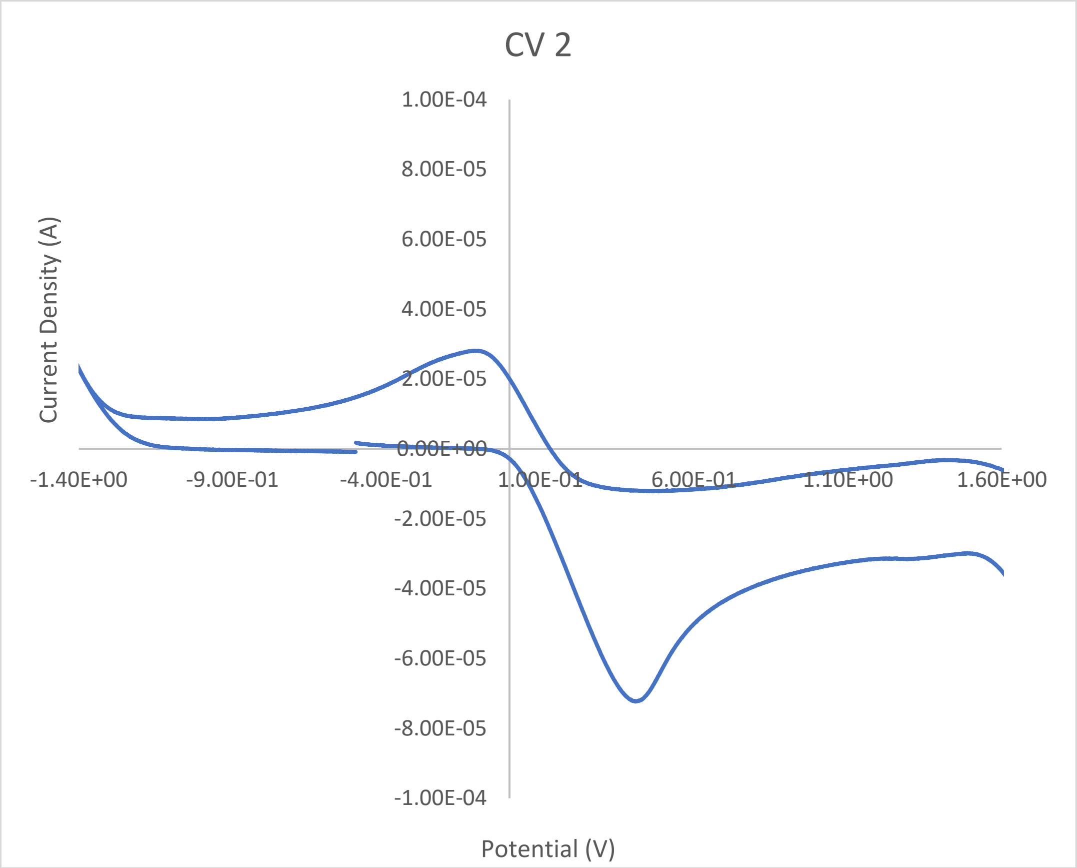

Potential Range from -0.8 V to 1.2

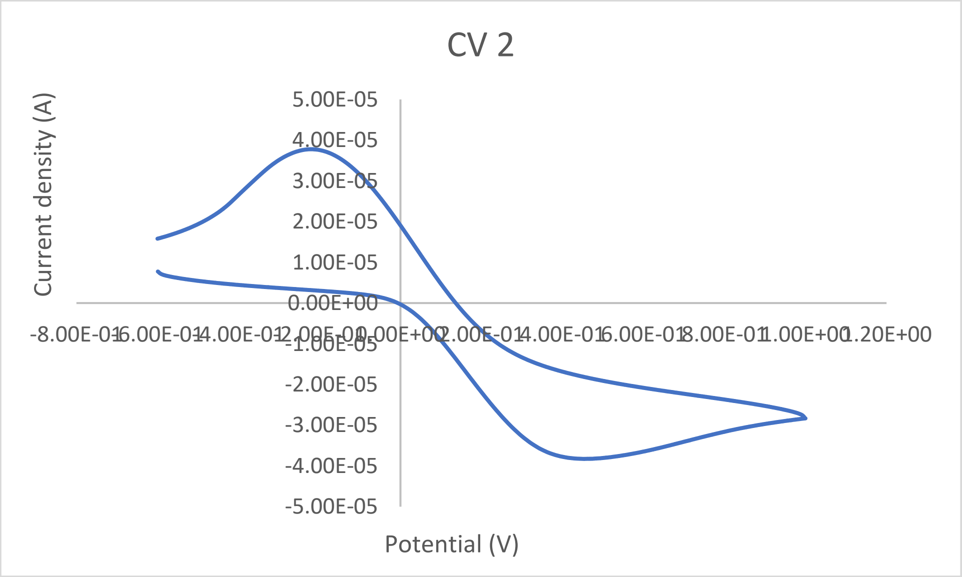

data for [$Ph_3PNNPPh_3$]

Potential Range from -1.7V to 2.8

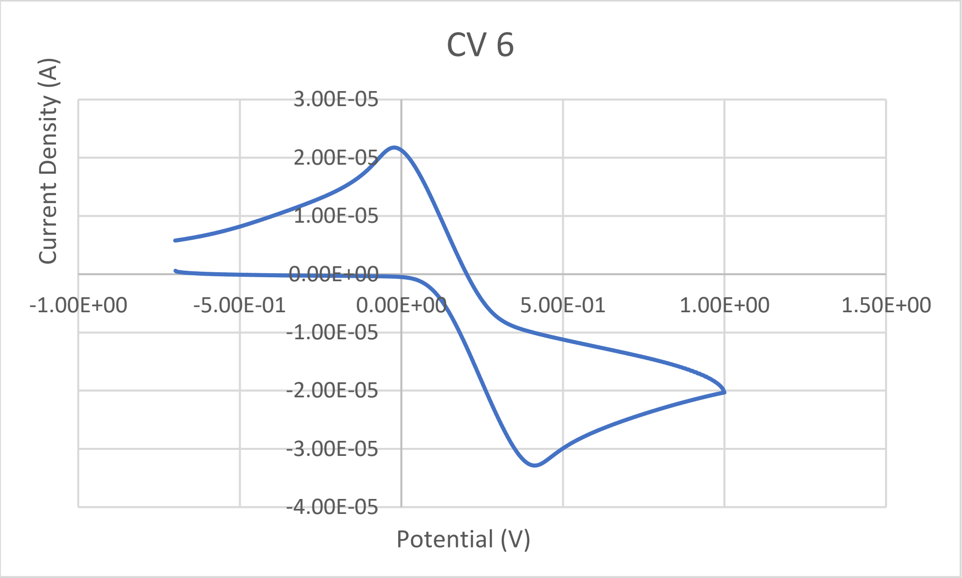

Potential Range from -0.8 V to 1.2

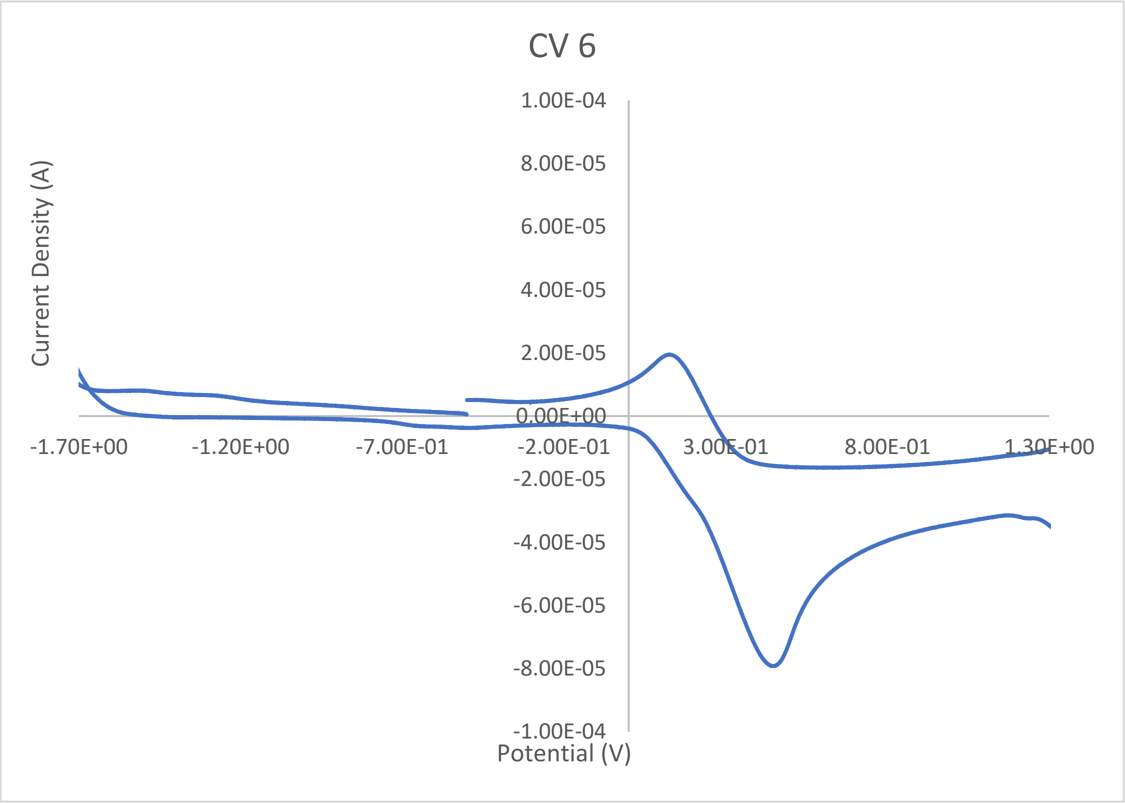

Computational Details

Supporting Information (Theory part)

1. **Computational details**
2. **Table S1.** TPSS-D3/def2-TZVP + COSMO computed lowest imaginary frequency (ImF), zero-point energies (ZPE), gas-phase enthalpic (Hc) and Gibbs free-energy (Gc) corrections; the COSMO-RS computed solvation enthalpic (Hsol) and Gibbs free-energy (Gsol) corrections in CH_2Cl_2 solution; TPSS-D3/def2-QZVP and PW6B95-D3/def2-QZVP single-point energies (TPSS-D3 and PW6B95-D3); the relative electronic energies (ΔE_T and ΔE_P) and Gibbs free-energies (ΔG_T and ΔG_P) at the TPSS-D3 and PW6B95-D3 levels. Each structure is labeled either by its molecular formula or a specific name in bold.
3. **Table S2.** The TPSS-D3/def2-TZVP + COSMO optimized atomic Cartesian coordinates (in Å) in CH_2Cl_2 solution. Each structure is labeled by the specific name (See also **Table S1**), followed by the number of atoms, the total energy, and the detailed atomic coordinates (in double-column text list).

Computational Details: The quantum chemical DFT calculations have been performed with the TURBOMOLE 7.4 suite of programs[5] The structures are fully optimized at the TPSS-D3/def2-TZVP + COSMO(CH_2Cl_2) level of theory, which combines the TPSS meta-GGA density functional[6] with the BJ-damped DFT-D3 dispersion correction[7] and the def2-TZVP basis set,[8] using the Conductor-like Screening Model (COSMO) continuum solvation model[9] for CH_2Cl_2 solvent (dielectric constant ε = 8.93 and solvent radius R_{solv} = 2.94 Å). The density-fitting RI-J approach[8a, 10] is used to accelerate the geometry optimization and numerical harmonic frequency calculations[11] in solution. The optimized structures are characterized by frequency analysis to identify the nature of located stationary points (no imaginary frequency for true minima and only one imaginary frequency for transition state) and to provide thermal corrections (at 298.15 K and 1 atm) according to the modified ideal gas–rigid rotor–harmonic oscillator model.[12] This choice of dispersion-corrected meta-GGA functional makes the efficient exploration of all potential reaction paths possible.

The final solvation free energies in CH_2Cl_2 are computed with the COSMO-RS solvation model[13] (parameter file: BP_TZVP_C30_1601.ctd) using the COSMOtherm program package[[14] on the above TPSS-D3 optimized structures, and corrected by +1.89 kcal·mol^{-1} to account for higher reference solute concentration of 1 mol·L^{-1} usually used in solution. To check the effects of the chosen DFT functional on the reaction energies and barriers, single-point calculations at the meta-GGA TPSS-D3[6] and hybrid-meta-GGA PW6B95-D3[15] levels are performed using a larger def2-QZVP basis set.[8b, 16] The final reaction Gibbs free energies (ΔG) are determined from the electronic single-point energies plus TPSS-D3 thermal corrections and COSMO-RS solvation free energies. In our discussion, the higher-level PW6B95-D3 Gibbs free energies (in kcal/mol, at 298.15 K and 1 mol/L standard state concentration) will be used in our discussion unless specified otherwise, since meta-GGA functionals usually underestimate reaction barriers that could be improved by using hybrid meta-GGA functionals.

Table S1. TPSS-D3/def2-TZVP + COSMO computed lowest imaginary frequency (ImF), zero-point energies (ZPE), gas-phase enthalpic (Hc) and Gibbs free-energy (Gc) corrections; the COSMO-RS computed solvation enthalpic (Hsol) and Gibbs free-energy (Gsol) corrections in CH_2Cl_2 solution; TPSS-D3/def2-QZVP and PW6B95-D3/def2-QZVP single-point energies (TPSS-D3 and PW6B95-D3); the relative electronic energies (ΔE_T and ΔE_P) and Gibbs free-energies (ΔG_T and ΔG_P) at the TPSS-D3 and PW6B95-D3 levels. Each structure is labeled either by its molecular formula or a specific name in bold. See also main-text Figure 7 for labellings.

Reactions	ImF	Zpe	Hc	Gc	Hsol	Gsol	TPSS	PW6B95	GP	E_T	E_P	*ΔG_P*	ΔG_T
in CH_2Cl_2 (1M)	cm^{-1}	kcal/mol	kcal/mol	kcal/mol	kcal/mol	kcal/mol	Eh	Eh		kcal/mol	kcal/mol	*kcal/mol*	kcal/mol
**2**	0	603.73	630.97	562.60	-29.91	-23.37	-2204.96480	-2207.11623	-2206.25392	0.00	0.00	*0.00*	0.00
**5**$^{\cdot+}$	0	618.33	642.29	581.28	-58.23	-49.61	-2204.81137	-2206.96201	-2206.11173	96.28	96.78	*89.22*	88.73
**5**$^{++}$	0	607.33	634.38	566.48	-143.63	-131.86	-2204.50339	-2206.64966	-2205.95403	289.54	292.78	188.18	184.94
**1**	0	344.68	367.40	307.00	-41.13	-30.61	-2183.18832	-2185.35727	-2184.91379	0.00	0.00	*0.00*	0.00
**6**$^{\cdot+}$	0	353.07	373.77	317.66	-59.90	-50.04	-2183.01384	-2185.18182	-2184.75232	109.49	110.10	*101.33*	100.72
**6**$^{++}$	0	346.69	369.59	307.50	-140.79	-128.46	-2182.70808	-2184.87144	-2184.58313	301.36	304.86	*207.50*	203.99
Cp_2Fe	0	104.65	110.60	83.88	-11.70	-8.26	-1651.22372	-1652.26455	-1652.14103	0.00	0.00	*0.00*	0.00
Cp_2Fe^+	0	106.67	112.57	85.85	-53.98	-48.41	-1650.98001	-1652.02772	-1651.96504	152.93	148.61	*110.44*	114.75

Table S2. The TPSS-D3/def2-TZVP + COSMO optimized atomic Cartesian coordinates (in Å) in CH_2Cl_2 solution. Each structure is labeled by the specific name (See also **Table S1**), followed by the number of atoms, the total energy, and the detailed atomic coordinates (in double-column text list).

1 : $Ph_3P{=}N{-}N{=}PPh_3$
70
Energy = -2183.095018170

P	1.7075222	-0.5116363	0.5065644
C	1.8675445	0.6956744	-0.8375953
N	0.2300277	-0.8166857	1.0621861
C	2.5667516	-1.9763681	-0.1591815
C	2.6559787	0.1027046	1.9137760
C	2.2292483	2.0268120	-0.5919766
C	1.4951189	0.3096782	-2.1358643
C	3.6947333	-1.9002143	-0.9869652
C	2.0577274	-3.2276865	0.2064288
C	1.9574232	0.6227875	3.0127998
C	4.0569340	0.1174883	1.9099075
H	2.4972651	2.3361793	0.4138946
C	2.2460430	2.9518775	-1.6337052
H	1.2118720	-0.7208784	-2.3220529
C	1.4960419	1.2420379	-3.1717763
H	4.0741823	-0.9312910	-1.3005109
C	4.3215156	-3.0673853	-1.4239061
C	2.6773676	-4.3943370	-0.2404269
H	1.1666981	-3.2602761	0.8272536
C	2.6573185	1.1492990	4.0976690
H	0.8728238	0.5943252	2.9960802
C	4.7534035	0.6537416	2.9933266
H	4.6042784	-0.2906076	1.0651902
H	2.5351942	3.9803200	-1.4376444
C	1.8788311	2.5614632	-2.9238172
H	1.1998102	0.9394690	-4.1726151
C	3.8132050	-4.3142713	-1.0504133
H	5.1999154	-3.0054729	-2.0604816
H	2.2743175	-5.3645697	0.0370048
H	2.1141531	1.5472650	4.9503994
C	4.0548335	1.1698226	4.0871213
H	5.8395775	0.6654901	2.9847994
H	1.8847163	3.2869261	-3.7326702
H	4.2977256	-5.2227870	-1.3976840
H	4.5985812	1.5859050	4.9306970
N	-0.5782376	-1.2253728	-0.1375124
P	-1.8316663	-0.2190391	-0.1769131
C	-1.4813928	1.5607006	-0.1916528
C	-2.9688732	-0.3950929	1.2379895
C	-2.7443433	-0.6122221	-1.6834335
C	-1.4954211	2.3040460	-1.3791932
C	-1.0668204	2.1741079	1.0021277
C	-2.8702962	-1.5741189	1.9853511

C	-3.9210242	0.5715858	1.5877645
C	-4.0794122	-0.2265631	-1.8608577
C	-2.0692322	-1.2782697	-2.7162855
H	-1.7944559	1.8295551	-2.3091249
C	-1.1281027	3.6480379	-1.3688341
H	-1.0529737	1.5945993	1.9191132
C	-0.6831542	3.5139531	1.0051588
H	-2.1060071	-2.2957627	1.7107103
C	-3.7243585	-1.7925388	3.0659284
C	-4.7836768	0.3471629	2.6608444
H	-3.9789667	1.5033974	1.0315050
C	-4.7320335	-0.4994600	-3.0633486
H	-4.6106271	0.2829483	-1.0622360
C	-2.7278200	-1.5575019	-3.9130379
H	-1.0377483	-1.5731259	-2.5535464
C	-0.7210170	4.2542417	-0.1778649
H	-1.1490755	4.2204332	-2.2917173
H	-0.3560844	3.9812246	1.9301540
C	-4.6856031	-0.8348428	3.3996571
H	-3.6391281	-2.7044460	3.6506998
H	-5.5258436	1.0953293	2.9256190
C	-4.0574260	-1.1644782	-4.0896811
H	-5.7665277	-0.1963257	-3.1973132
H	-2.2043640	-2.0783951	-4.7100428
H	-0.4272199	5.3002980	-0.1741600
H	-5.3528400	-1.0050681	4.2402543
H	-4.5674304	-1.3775658	-5.0250431

2 : $Cy_3P{=}N{-}N{=}PCy_3$

106

Energy = -2204.840803301

P	-1.9284793	-0.0774831	-0.0156711
N	-0.4382322	0.0489815	-0.5788055
N	0.5486608	-0.0422739	0.5651743
P	1.9841480	0.1702958	-0.1064786
C	-2.4798273	1.3586545	1.0294767
C	-3.9823211	1.6043926	1.2609147
C	-1.7013241	1.3679446	2.3635883
H	-2.0981933	2.2003970	0.4292785
C	-4.1981141	2.9223474	2.0226296
H	-4.4181608	0.7783555	1.8349616
H	-4.5175211	1.6459949	0.3066404
C	-1.9275198	2.6872788	3.1123881
H	-0.6397524	1.1930125	2.1599497
H	-2.0509727	0.5370127	2.9918716
C	-3.4214961	2.9470673	3.3457585
H	-5.2692344	3.0754055	2.2052707
H	-3.8585490	3.7558041	1.3906472
H	-1.3898244	2.6714131	4.0688985
H	-1.5032953	3.5114373	2.5202362
H	-3.5704022	3.9085145	3.8528257
H	-3.8211982	2.1686455	4.0121636

C	-2.0941119	-1.6087832	1.0046265
C	-3.3461138	-1.8601288	1.8567013
C	-1.7580449	-2.8312021	0.1290737
H	-1.2341905	-1.4588193	1.6794271
C	-3.1609965	-3.1218467	2.7165108
H	-4.2247284	-1.9881577	1.2115634
H	-3.5501541	-1.0034914	2.5063470
C	-1.5683552	-4.0804196	0.9974338
H	-2.5752723	-3.0115011	-0.5843913
H	-0.8584209	-2.6123790	-0.4541182
C	-2.8100566	-4.3458665	1.8597392
H	-4.0705882	-3.3134938	3.2995893
H	-2.3495395	-2.9421737	3.4365664
H	-1.3498703	-4.9502708	0.3650449
H	-0.6966432	-3.9293628	1.6501361
H	-2.6516510	-5.2224113	2.5002159
H	-3.6599084	-4.5801623	1.2018349
C	2.1533363	1.7866943	-1.0091393
C	1.2530796	2.8196781	-0.3046450
C	3.5750482	2.3481295	-1.1854052
H	1.7226028	1.5883379	-2.0018826
C	1.2256156	4.1428370	-1.0792325
H	1.6346806	2.9977286	0.7113261
H	0.2506056	2.3951508	-0.2062026
C	3.5455368	3.6701109	-1.9690940
H	4.0148625	2.5335717	-0.1956316
H	4.2265454	1.6307225	-1.6944846
C	2.6384560	4.7034171	-1.2885818
H	0.6012957	4.8738187	-0.5493684
H	0.7548233	3.9712342	-2.0584367
H	4.5641921	4.0652960	-2.0711538
H	3.1738301	3.4735667	-2.9850654
H	2.6026189	5.6258128	-1.8817383
H	3.0682073	4.9680768	-0.3112658
C	-2.9396797	-0.1435466	-1.5457144
C	-4.3625110	-0.7155162	-1.4202522
C	-2.9416023	1.2260025	-2.2561994
H	-2.3476036	-0.8327888	-2.1652888
C	-5.0286526	-0.8029041	-2.8016381
H	-4.9717134	-0.0807482	-0.7664029
H	-4.3333932	-1.7087828	-0.9609092
C	-3.6187214	1.1256018	-3.6293484
H	-3.4845120	1.9575544	-1.6422290
H	-1.9096951	1.5784854	-2.3506459
C	-5.0391465	0.5585745	-3.5086132
H	-6.0506258	-1.1869302	-2.6962735
H	-4.4759020	-1.5254737	-3.4191847
H	-3.6406347	2.1128448	-4.1072113
H	-3.0204502	0.4681727	-4.2763956
H	-5.4991874	0.4668728	-4.5001057
H	-5.6594332	1.2598613	-2.9315620
C	3.1957732	0.1617578	1.2724830

C	2.7092696	-0.7076192	2.4487496
C	4.6365711	-0.2196063	0.8813300
H	3.2085669	1.2083651	1.6165713
C	3.6477662	-0.5500209	3.6522421
H	2.6906019	-1.7585883	2.1333628
H	1.6803490	-0.4354842	2.6965631
C	5.5762571	-0.0646071	2.0861366
H	4.6541900	-1.2629958	0.5424615
H	4.9937733	0.3918846	0.0477388
C	5.0962727	-0.8973238	3.2822699
H	3.3040303	-1.1851942	4.4782788
H	3.6044478	0.4895902	4.0091250
H	6.5949812	-0.3559035	1.8023760
H	5.6144459	0.9959580	2.3739846
H	5.7598702	-0.7426082	4.1420305
H	5.1534484	-1.9641850	3.0216303
C	2.2974902	-1.1236300	-1.3921745
C	3.4408965	-0.9613831	-2.4031364
C	2.3011886	-2.5256659	-0.7577674
H	1.3405947	-1.0211452	-1.9345724
C	3.3636641	-2.0584991	-3.4787855
H	4.4105548	-1.0250542	-1.8919486
H	3.3926076	0.0220314	-2.8828695
C	2.2107472	-3.6079078	-1.8406612
H	3.2276379	-2.6727248	-0.1850561
H	1.4676300	-2.6001636	-0.0515598
C	3.3510983	-3.4620516	-2.8579201
H	4.2047000	-1.9588127	-4.1767040
H	2.4427633	-1.9157476	-4.0623164
H	2.2335848	-4.6048797	-1.3825534
H	1.2458350	-3.5131348	-2.3590032
H	3.2625870	-4.2220757	-3.6441213
H	4.3099543	-3.6392051	-2.3487668

5$^{\cdot+}$: radical cation of $Cy_3P{=}N{-}N{=}PCy_3$
106
Energy = -2204.726566692

P	-2.0110201	-0.1062689	0.0377649
N	-0.4472686	0.0624979	-0.5457941
N	0.4885202	-0.0572101	0.4458585
P	2.0198741	0.1992024	-0.1845471
C	-2.4771445	1.4450468	0.9004146
C	-3.9833253	1.7023111	1.1072047
C	-1.6954848	1.5778927	2.2259440
H	-2.0990691	2.2115926	0.2058177
C	-4.1918171	3.0911046	1.7313846
H	-4.4093366	0.9352880	1.7631482
H	-4.5165025	1.6455875	0.1538027
C	-1.9179591	2.9700903	2.8313302
H	-0.6312882	1.3876942	2.0552483
H	-2.0501002	0.8166257	2.9319272
C	-3.4110950	3.2491907	3.0417602

H	-5.2621888	3.2539740	1.8998854
H	-3.8588295	3.8548471	1.0149368
H	-1.3752583	3.0459963	3.7802341
H	-1.4941742	3.7274178	2.1567074
H	-3.5551696	4.2578770	3.4449544
H	-3.8074269	2.5457326	3.7873468
C	-2.0530964	-1.5473882	1.1584226
C	-3.2985690	-1.7313515	2.0431356
C	-1.7272262	-2.8310448	0.3646657
H	-1.1881333	-1.3281739	1.8053511
C	-3.0900978	-2.9225703	2.9913780
H	-4.1809733	-1.9108791	1.4181777
H	-3.4948751	-0.8263992	2.6254303
C	-1.5231908	-4.0082997	1.3264975
H	-2.5555509	-3.0612825	-0.3175723
H	-0.8342556	-2.6741069	-0.2487810
C	-2.7517927	-4.2064037	2.2231321
H	-3.9911311	-3.0654818	3.5981312
H	-2.2700894	-2.6855775	3.6836949
H	-1.3154443	-4.9185026	0.7528397
H	-0.6403907	-3.8112530	1.9507273
H	-2.5785616	-5.0298136	2.9250720
H	-3.6104287	-4.4920789	1.5992736
C	2.1409956	1.8996686	-0.8756766
C	1.2877443	2.8773267	-0.0394301
C	3.5814311	2.4334176	-1.0154452
H	1.6898409	1.8131571	-1.8750575
C	1.2924734	4.2749853	-0.6730532
H	1.6943948	2.9343432	0.9797218
H	0.2658338	2.5005503	0.0385077
C	3.5726343	3.8267855	-1.6622755
H	4.0337379	2.5074416	-0.0178788
H	4.2021208	1.7519474	-1.6038289
C	2.7176937	4.8095505	-0.8542633
H	0.7027346	4.9573579	-0.0500027
H	0.7948668	4.2226198	-1.6515601
H	4.6023148	4.1919798	-1.7458432
H	3.1751231	3.7451989	-2.6833234
H	2.6954543	5.7872823	-1.3485511
H	3.1763592	4.9588849	0.1332792
C	-2.9539305	-0.3497343	-1.5019775
C	-4.3863024	-0.8937120	-1.3415878
C	-2.9202967	0.9346546	-2.3599367
H	-2.3518380	-1.1129084	-2.0185894
C	-5.0117030	-1.1354522	-2.7232674
H	-5.0012674	-0.1796959	-0.7832121
H	-4.3789730	-1.8264382	-0.7691377
C	-3.5600540	0.6741104	-3.7301106
H	-3.4765527	1.7300198	-1.8476858
H	-1.8858401	1.2752064	-2.4722152
C	-4.9879871	0.1345881	-3.5825358
H	-6.0388618	-1.4951132	-2.5976126

H	-4.4520925	-1.9314490	-3.2341006
H	-3.5573493	1.6007310	-4.3149218
H	-2.9483960	-0.0552898	-4.2791109
H	-5.4178276	-0.0709741	-4.5692372
H	-5.6181944	0.9024836	-3.1119846
C	3.1481965	0.0212367	1.2403014
C	2.6296151	-0.9913154	2.2852406
C	4.6034528	-0.3041265	0.8392875
H	3.1364923	1.0205231	1.7040174
C	3.5430370	-0.9851141	3.5185519
H	2.6176514	-1.9945804	1.8421379
H	1.6001810	-0.7477647	2.5576517
C	5.5062281	-0.2986827	2.0808757
H	4.6330045	-1.2975055	0.3756948
H	4.9786923	0.4073309	0.0990880
C	5.0002910	-1.2813481	3.1436359
H	3.1774257	-1.7208148	4.2439671
H	3.4838487	-0.0002800	4.0030639
H	6.5313383	-0.5468502	1.7842577
H	5.5298838	0.7164788	2.5009810
H	5.6377631	-1.2352497	4.0338019
H	5.0711499	-2.3055471	2.7510012
C	2.2523840	-1.0109576	-1.5388772
C	3.4110950	-0.7656698	-2.5204375
C	2.2503428	-2.4584267	-1.0068551
H	1.3024757	-0.8520865	-2.0783417
C	3.3418452	-1.7795833	-3.6739139
H	4.3711499	-0.8676437	-2.0009166
H	3.3622169	0.2504477	-2.9239192
C	2.1778438	-3.4510628	-2.1742949
H	3.1689307	-2.6422060	-0.4363664
H	1.4101547	-2.6049293	-0.3203060
C	3.3303674	-3.2247546	-3.1605871
H	4.1899785	-1.6188752	-4.3488310
H	2.4283146	-1.5932278	-4.2556980
H	2.1993678	-4.4754574	-1.7859097
H	1.2186901	-3.3232415	-2.6955184
H	3.2505895	-3.9220739	-4.0020517
H	4.2833525	-3.4384883	-2.6562712

5^{++} : dication of Cy_3P=N–N=PCy_3
106
Energy = -2204.533364383

P	-2.0870042	-0.1504117	0.1873929
N	-0.4212418	0.0862214	-0.4014847
N	0.4534866	0.2689932	0.4643189
P	2.1068999	0.4316758	-0.2291395
C	-2.7210425	1.5578101	0.2552940
C	-4.2623672	1.6588469	0.1887239
C	-2.1614656	2.2762499	1.5011687
H	-2.3034180	2.0245012	-0.6492445
C	-4.6659233	3.1423839	0.1887772

H	-4.7058429	1.1490727	1.0509991
H	-4.6410921	1.1725346	-0.7141437
C	-2.5713397	3.7555196	1.4690215
H	-1.0719409	2.1790658	1.5531204
H	-2.5674528	1.8086000	2.4049059
C	-4.0967913	3.8921023	1.3982833
H	-5.7588565	3.2057772	0.1754847
H	-4.3028455	3.6071191	-0.7373458
H	-2.1788617	4.2524882	2.3619567
H	-2.1122575	4.2390399	0.5965258
H	-4.3756588	4.9494988	1.3454681
H	-4.5383436	3.4885747	2.3194347
C	-1.9827670	-0.9922197	1.7912358
C	-3.2955069	-1.0025942	2.6065282
C	-1.4276695	-2.4227618	1.6052572
H	-1.2353306	-0.3888869	2.3310307
C	-3.0345650	-1.6563927	3.9721034
H	-4.0619979	-1.5675276	2.0655740
H	-3.6758506	0.0131757	2.7450735
C	-1.1759614	-3.0586243	2.9793404
H	-2.1576977	-3.0267824	1.0543438
H	-0.5047285	-2.4150138	1.0151199
C	-2.4555218	-3.0682561	3.8238388
H	-3.9724090	-1.6824957	4.5360128
H	-2.3343521	-1.0278444	4.5388594
H	-0.7972810	-4.0758908	2.8379753
H	-0.3935500	-2.4913398	3.5022417
H	-2.2483714	-3.4926951	4.8114032
H	-3.1993383	-3.7183148	3.3437564
C	2.4799175	2.2173237	-0.1363694
C	1.8212133	2.8880353	1.0894635
C	4.0060863	2.4746476	-0.1415456
H	2.0379401	2.6274113	-1.0552918
C	2.1136472	4.3953882	1.0846071
H	2.2112240	2.4352631	2.0098320
H	0.7412588	2.7220052	1.0774980
C	4.2728069	3.9877851	-0.1566877
H	4.4442965	2.0414462	0.7658796
H	4.4855472	1.9995187	-1.0015245
C	3.6188455	4.6788655	1.0440466
H	1.6591832	4.8447305	1.9737748
H	1.6293099	4.8474012	0.2088314
H	5.3553653	4.1507182	-0.1555764
H	3.8814895	4.4117083	-1.0906961
H	3.7954211	5.7584155	0.9991573
H	4.0834590	4.3144504	1.9702254
C	-2.7185864	-1.1680132	-1.1755570
C	-4.0991917	-1.8039690	-0.8982415
C	-2.7024820	-0.3734109	-2.5010835
H	-1.9761714	-1.9796353	-1.2437944
C	-4.4884438	-2.7047098	-2.0800692
H	-4.8509620	-1.0185839	-0.7662798

H	-4.0752156	-2.3910447	0.0244319
C	-3.1036533	-1.2979454	-3.6595694
H	-3.4151123	0.4573798	-2.4366598
H	-1.7118557	0.0571430	-2.6768991
C	-4.4734227	-1.9383103	-3.4071913
H	-5.4796745	-3.1279039	-1.8888528
H	-3.7821272	-3.5438329	-2.1326809
H	-3.1111544	-0.7197143	-4.5890861
H	-2.3443842	-2.0840593	-3.7727201
H	-4.7286046	-2.6132706	-4.2306248
H	-5.2420231	-1.1537464	-3.3850424
C	3.1070121	-0.5358922	0.9468229
C	2.3529696	-1.7654777	1.5027579
C	4.4737919	-0.9403904	0.3456832
H	3.2791272	0.1695155	1.7744206
C	3.2172099	-2.4762138	2.5539341
H	2.1246394	-2.4581216	0.6844936
H	1.4052259	-1.4510843	1.9490250
C	5.3161006	-1.6503007	1.4157883
H	4.3074467	-1.6220093	-0.4964479
H	5.0088527	-0.0694827	-0.0409667
C	4.5838779	-2.8711420	1.9836041
H	2.6796826	-3.3588966	2.9158802
H	3.3552075	-1.8066214	3.4132572
H	6.2727282	-1.9440864	0.9721389
H	5.5392439	-0.9415769	2.2243495
H	5.1910143	-3.3447310	2.7616907
H	4.4448768	-3.6148095	1.1869723
C	2.0083334	-0.1599867	-1.9442156
C	3.2109297	0.2489777	-2.8247302
C	1.7121674	-1.6725236	-2.0400483
H	1.1212441	0.3857191	-2.3056572
C	2.9395843	-0.1661339	-4.2791248
H	4.1216241	-0.2402596	-2.4627756
H	3.3706919	1.3299646	-2.7753772
C	1.4477906	-2.0537945	-3.5033379
H	2.5731014	-2.2389224	-1.6674873
H	0.8526014	-1.9429978	-1.4183754
C	2.6299412	-1.6626466	-4.3972919
H	3.8101529	0.0965474	-4.8884341
H	2.0903106	0.4164012	-4.6609198
H	1.2550640	-3.1296997	-3.5633998
H	0.5385735	-1.5419683	-3.8485424
H	2.4128206	-1.9188283	-5.4392974
H	3.5141404	-2.2436528	-4.1018445

$\mathbf{6}^{\cdot+}$: radical cation of $Ph_3P{=}N{-}N{=}PPh_3$

70

Energy = -2182.953860785

P	1.9056252	-0.3272605	0.4873162
C	2.2708015	1.1402031	-0.4819955
N	0.2627747	-0.3416560	0.7922110

C	2.5001188	-1.7676184	-0.4047717
C	2.6761531	-0.2144176	2.0975294
C	2.9253207	2.2405529	0.0881933
C	1.7531494	1.2220244	-1.7868891
C	3.5091483	-1.6643176	-1.3721998
C	1.9398338	-3.0170780	-0.0956025
C	1.9477407	0.2889789	3.1866993
C	4.0143373	-0.6029525	2.2568291
H	3.3155959	2.1789011	1.0994771
C	3.0715817	3.4144235	-0.6496597
H	1.2307826	0.3734317	-2.2175805
C	1.8981189	2.4004527	-2.5115189
H	3.9351583	-0.6958174	-1.6151465
C	3.9605871	-2.8119052	-2.0230304
C	2.3926149	-4.1564972	-0.7541604
H	1.1513288	-3.0897976	0.6474053
C	2.5650545	0.4002864	4.4309176
H	0.9101217	0.5743640	3.0505497
C	4.6231877	-0.4827446	3.5042095
H	4.5724140	-1.0021665	1.4147274
H	3.5841049	4.2651986	-0.2112532
C	2.5566267	3.4951548	-1.9444327
H	1.4929088	2.4683716	-3.5161886
C	3.4025639	-4.0540273	-1.7153421
H	4.7438462	-2.7346632	-2.7707871
H	1.9575384	-5.1235890	-0.5216920
H	2.0050826	0.7853933	5.2776733
C	3.8996343	0.0184264	4.5890480
H	5.6583599	-0.7842763	3.6299737
H	2.6654983	4.4137562	-2.5131721
H	3.7527054	-4.9453565	-2.2274583
H	4.3759585	0.1081904	5.5607774
N	-0.4435395	-0.7203673	-0.3041435
P	-1.9665493	-0.0333612	-0.3212580
C	-1.7873678	1.6878106	-0.8029896
C	-2.8220972	-0.1019753	1.2557809
C	-2.8688290	-0.9369133	-1.5735952
C	-2.1803134	2.1289483	-2.0739332
C	-1.1077316	2.5563488	0.0693881
C	-2.6753195	-1.2564745	2.0407970
C	-3.6327997	0.9539264	1.6946497
C	-4.2712342	-0.9390580	-1.5554401
C	-2.1699716	-1.6155450	-2.5842962
H	-2.6961803	1.4539325	-2.7499807
C	-1.9044078	3.4383245	-2.4653562
H	-0.7885200	2.2045143	1.0454857
C	-0.8312495	3.8585533	-0.3338166
H	-2.0383115	-2.0673217	1.7004525
C	-3.3410035	-1.3494444	3.2594981
C	-4.3007082	0.8497133	2.9142314
H	-3.7382386	1.8488970	1.0893764
C	-4.9724494	-1.6168957	-2.5504577

H -4.8092473 -0.4214229 -0.7664302
C -2.8813583 -2.2938594 -3.5719036
H -1.0851986 -1.6144587 -2.5788352
C -1.2292559 4.2995517 -1.5990101
H -2.2137319 3.7828706 -3.4473281
H -0.3004647 4.5284904 0.3352586
C -4.1537456 -0.2984596 3.6946174
H -3.2249526 -2.2382601 3.8720114
H -4.9314515 1.6650675 3.2547245
C -4.2784118 -2.2926139 -3.5570147
H -6.0578624 -1.6219132 -2.5367840
H -2.3458671 -2.8237398 -4.3538018
H -1.0096083 5.3164454 -1.9101694
H -4.6713231 -0.3748013 4.6462496
H -4.8276009 -2.8222466 -4.3297427

6^{++} : dication of $Ph_3P=N–N=PPh_3$
70
Energy = -2182.759069837
P 2.0909425 -0.1546539 0.3622191
C 2.7074175 1.4924763 0.1108842
N 0.3340923 0.1604408 0.4970524
C 2.4345072 -1.2600708 -0.9778474
C 2.4319747 -0.8340663 1.9632395
C 3.5590830 2.0829225 1.0568238
C 2.2887386 2.2033326 -1.0306231
C 3.2821610 -0.8809505 -2.0310489
C 1.7874532 -2.5110239 -0.9878713
C 1.8216791 -0.2647339 3.0978790
C 3.3042088 -1.9284466 2.0834768
H 3.8728835 1.5291584 1.9356985
C 4.0039042 3.3857788 0.8473766
H 1.6254687 1.7435472 -1.7577272
C 2.7373626 3.5047670 -1.2218767
H 3.7849432 0.0801495 -2.0147611
C 3.4836542 -1.7607584 -3.0912507
C 1.9942735 -3.3753076 -2.0563172
H 1.1366575 -2.8036505 -0.1692192
C 2.0914112 -0.8028172 4.3512445
H 1.1466781 0.5784630 2.9962496
C 3.5658432 -2.4505784 3.3469094
H 3.7715389 -2.3591453 1.2039447
H 4.6701232 3.8476744 1.5682173
C 3.5928619 4.0931199 -0.2847139
H 2.4238701 4.0595563 -2.1000760
C 2.8398128 -2.9998463 -3.1050606
H 4.1446775 -1.4792040 -3.9040706
H 1.5010708 -4.3415902 -2.0704558
H 1.6263895 -0.3731367 5.2323764
C 2.9599620 -1.8915057 4.4746992
H 4.2415962 -3.2929508 3.4500144
H 3.9398205 5.1098819 -0.4389681

H	3.0005833	-3.6800816	-3.9354845
H	3.1657040	-2.3057717	5.4566386
N	-0.3379736	-0.1735042	-0.4888558
P	-2.0804002	0.2250512	-0.3930670
C	-2.1722154	1.5930542	-1.5228118
C	-2.5461162	0.6337130	1.2657496
C	-2.7991758	-1.2791707	-0.9946496
C	-2.9354147	1.4940456	-2.6960912
C	-1.4299222	2.7554374	-1.2370956
C	-2.2782387	-0.3059528	2.2801409
C	-3.1140902	1.8806211	1.5727963
C	-3.9500271	-1.7965419	-0.3774716
C	-2.2035860	-1.9384020	-2.0877139
H	-3.5006670	0.5928053	-2.9100823
C	-2.9654141	2.5719517	-3.5772111
H	-0.8370017	2.8256991	-0.3295957
C	-1.4662415	3.8200575	-2.1297597
H	-1.8437014	-1.2714436	2.0388929
C	-2.5817685	0.0114204	3.5986600
C	-3.4177258	2.1809382	2.8980645
H	-3.3260470	2.5978998	0.7870368
C	-4.5052781	-2.9777099	-0.8612222
H	-4.4031938	-1.2807001	0.4627742
C	-2.7695899	-3.1197337	-2.5539403
H	-1.3130505	-1.5339055	-2.5569352
C	-2.2325577	3.7273532	-3.2961768
H	-3.5601086	2.5087144	-4.4822783
H	-0.9011398	4.7214984	-1.9168896
C	-3.1492832	1.2521737	3.9057690
H	-2.3808093	-0.7079291	4.3856391
H	-3.8655613	3.1383624	3.1423245
C	-3.9158011	-3.6369695	-1.9426810
H	-5.3965406	-3.3825653	-0.3937191
H	-2.3190338	-3.6368920	-3.3946715
H	-2.2569666	4.5617162	-3.9898841
H	-3.3873090	1.4936856	4.9368666
H	-4.3519212	-4.5595259	-2.3125921

Cp_2Fe : neutral
21
Energy = -1651.176296280

Fe	-0.0000975	-0.0008070	-0.0000097
C	-0.0004144	1.2196357	1.6292815
C	0.0011722	1.2195524	-1.6293702
C	0.7160811	-0.9864981	1.6312696
C	-0.7167260	-0.9861156	-1.6313366
C	0.7167708	-0.9868890	-1.6306792
C	-0.7174153	-0.9863539	1.6308353
C	1.1604971	0.3762573	-1.6295263
C	-1.1602642	0.3770697	1.6296835
C	1.1592645	0.3768223	1.6302585
C	-1.1590392	0.3774804	-1.6304763

H	-0.0002984	2.3013661	1.6027654
H	0.0017388	2.3012808	-1.6029388
H	1.3518394	-1.8617350	1.6065097
H	-1.3530478	-1.8609488	-1.6066392
H	1.3521432	-1.8623950	-1.6054312
H	-1.3533507	-1.8614571	1.6057156
H	2.1894918	0.7099302	-1.6033485
H	-2.1890568	0.7113993	1.6036894
H	2.1881406	0.7109158	1.6047612
H	-2.1877058	0.7122323	-1.6051520

$Cp_2Fe^{\cdot+}$: radical cation of Cp_2Fe

21

Energy = -1650.993359454

Fe	-0.0000007	-0.0000004	-0.1100419
C	-0.0000116	-1.7633347	-1.3154320
C	0.0000117	1.7633331	-1.3154335
C	0.7180572	-1.6491713	0.8784604
C	-0.7180560	1.6491852	0.8784589
C	0.7180898	1.6491560	0.8784615
C	-0.7180904	-1.6491690	0.8784609
C	1.1534725	1.7178619	-0.4834928
C	-1.1534699	-1.7178875	-0.4834907
C	1.1534457	-1.7178903	-0.4834877
C	-1.1534432	1.7179136	-0.4834870
H	-0.0000221	-1.7650645	-2.3973557
H	0.0000193	1.7650640	-2.3973571
H	1.3567946	-1.5971443	1.7492475
H	-1.3567985	1.5971647	1.7492429
H	1.3568322	1.5971337	1.7492499
H	-1.3568338	-1.5971537	1.7492488
H	2.1796289	1.6966787	-0.8247875
H	-2.1796246	-1.6967128	-0.8247914
H	2.1796100	-1.6967342	-0.8247637
H	-2.1796108	1.6967718	-0.8247546

References

[1] R. Appel, R. Schöllhorn, *Angew. Chem. Int . Ed.* **1964**, *3*, 805.
[2] M. M. Burgoyne, T. M. MacDougall, Z. N. Haines, J. W. Conrad, L. A. Calhoun, A. Decken, C. A. Dyker, *Org. Biomol. Chem.* **2019**, *17*, 9726–9733.
[3] Ł. Kapuśniak, P. N. Plessow, D. Trzybiński, K. Woźniak, P. Hofmann, P. I. Jolly, *Organometallics* **2021**, *40*, 693–701.
[4] H. Jacobsen, H. Berke, S. Döring, G. Kehr, G. Erker, R. Fröhlich , O. Meyer, *Organometallics* **1999**, *18*, 1724–1735.
[5] TURBOMOLE V7.0, 2015, a development of University of Karlsruhe and Forschungszentrum Karlsruhe GmbH, 1989-2007, TURBOMOLE GmbH, since 2007; available from http://www.turbomole.com.,
[6] J. Tao, J. P. Perdew, V. N. Staroverov, G. E. Scuseria, *Phys. Rev. Lett.* **2003**, *91*, 146401.
[7] (a) S. Grimme, J. Antony, S. Ehrlich, H. Krieg, *J. Chem. Phys.* **2010**, *132*, 154104-154119; (b) S. Grimme, S. Ehrlich, L. Goerigk, *J. Comp. Chem.* **2011**, *32*, 1456-1465.
[8] (a) F. Weigend, M. Häser, H. Patzelt, R. Ahlrichs, *Chem. Phys. Lett.* **1998**, *294*, 143-152; (b) F. Weigend, R. Ahlrichs, *phys. Chem. Chem. Phys.* **2005**, *7*, 3297-3305.
[9] A. Klamt, G. Schüürmann, *J. Chem. Soc. Perkin Trans.* **1993**, 799-805.
[10] (a) K. Eichkorn, F. Weigend, O. Treutler, R. Ahlrichs, *Theo. Chem. Accts* **1997**, *97*, 119-124; (b) F. Weigend, *phys. Chem. Chem. Phys.* **2006**, *8*, 1057-1065.
[11] P. Deglmann, K. May, F. Furche, R. Ahlrichs, *Chem. Phys. Lett.* **2004**, *384*, 103-107.
[12] S. Grimme, *Chem. Eur. J.* **2012**, *18*, 9955-9964.
[13] F. Eckert, A. Klamt, *AIChE Journal* **2002**, *48*, 369-385.
[14] COSMOtherm, Version C3.0, Release 16.01; COSMOlogic GmbH & Co. KG, Leverkusen, Germany 2015.
[15] Y. Zhao, D. G. Truhlar, *J. Phys. Chem.* **2005**, *109*, 5656-5667.
[16] F. Weigend, F. Furche, R. Ahlrichs, *J. Chem. Phys.* **2003**, *119*, 12753-12762.
